# A longitudinal examination of objective neighborhood walkability, body mass index, and waist circumference: the REasons for Geographic And Racial Differences in Stroke study

**DOI:** 10.1186/s12966-022-01247-7

**Published:** 2022-02-12

**Authors:** Ian-Marshall Lang, Cathy L. Antonakos, Suzanne E. Judd, Natalie Colabianchi

**Affiliations:** 1 School of Kinesiology, University of Michigan, 830 North University Avenue, Ann Arbor, MI 48109 USA; 2 Department of Biostatistics, University of Alabama at Birmingham, 1665 University Boulevard, Birmingham, Alabama, 35233 USA; 3 Institute for Social Research, University of Michigan, 426 Thompson Street, Ann Arbor, MI 48104 USA

**Keywords:** Neighborhood walkability, Walk Score®, Body mass index, Waist circumference, Obesity

## Abstract

**Background:**

Studies have shown neighborhood walkability is associated with obesity. To advance this research, study designs involving longer follow-up, broader geographic regions, appropriate neighborhood characterization, assessment of exposure length and severity, and consideration of stayers and movers are needed. Using a cohort spanning the conterminous United States, this study examines the longitudinal relationship between a network buffer-derived, duration-weighted neighborhood walkability measure and two adiposity-related outcomes.

**Methods:**

This study included 12,846 Black/African American and White adults in the REasons for Geographic And Racial Differences in Stroke study. Body mass index (BMI) and waist circumference (WC) were assessed at baseline and up to 13.3 years later (M (SD) = 9.4 (1.0) years). BMI and WC were dichotomized. Walk Score® was duration-weighted based on time at each address and categorized as Very Car-Dependent, Car-Dependent, Somewhat Walkable, Very Walkable, and Walker’s Paradise. Unadjusted and adjusted logistic regression models tested each neighborhood walkability-adiposity association. Adjusted models controlled for demographics, health factors, neighborhood socioeconomic status, follow-up time, and either baseline BMI or baseline WC. Adjusted models also tested for interactions. Post-estimation Wald tests examined whether categorical variables had coefficients jointly equal to zero. Orthogonal polynomial contrasts tested for a linear trend in the neighborhood walkability-adiposity relationships.

**Results:**

The odds of being overweight/obese at follow-up were lower for residents with duration-weighted Walk Score® values in the Walker’s Paradise range and residents with values in the Very Walkable range compared to residents with values in the Very Car-Dependent range. Residents with duration-weighted Walk Score® values classified as Very Walkable had significantly lower odds of having a moderate-to-high risk WC at follow-up relative to those in the Very Car-Dependent range. For both outcomes, the effects were small but meaningful. The negative linear trend was significant for BMI but not WC.

**Conclusion:**

People with cumulative neighborhood walkability scores in the Walker’s Paradise range were less likely to be overweight/obese independent of other factors, while people with scores in the Very Walkable range were less likely to be overweight/obese and less likely to have a moderate-to-high risk WC. Addressing neighborhood walkability is one approach to combating obesity.

**Supplementary Information:**

The online version contains supplementary material available at 10.1186/s12966-022-01247-7.

## Background

Obesity is a worldwide problem contributing to poorer quality of life and decreased life expectancy in low-, middle-, and high-income countries [1]. These impacts on morbidity and mortality have been well studied using various measures of adiposity including body mass index (BMI), waist circumference (WC), waist-to-height ratio, and waist-to-hip ratio [1, 2]. This research has highlighted the value of general measures of adiposity (i.e., BMI) as well as measures of adiposity in specific regions of the body (i.e., WC) given the differential and magnified impact abdominal adiposity has on health [3].

Public health researchers have found disparities in obesity incidence, prevalence, and long term sequelae, with differences patterned by underlying social phenomena and built environment (BE) characteristics [4]. Across various countries and settings, researchers have pointed to neighborhood walkability as one contributing BE factor. However, there is a need for stronger study designs to strengthen the evidence supporting the connection between neighborhood walkability and obesity.

A recent review of studies on neighborhood walkability and obesity among adults in high-income countries found cross-sectional study designs made up the majority of studies reporting an inverse relationship between objective walkability indices and markers of overweight/obesity [5]. No longitudinal studies in the review reported significant findings. However, a systematic review and meta-analysis of longitudinal studies examining the relationship between BE characteristics and cardio-metabolic health (including independent analyses of obesity outcomes) found strong evidence supporting the neighborhood walkability-obesity relationship [6]. Despite this finding, Chandrabose et al. [6] highlight the strength of association is weakened (though remains statistically significant) in studies of objective walkability. Although these two reviews differ in purpose and article inclusion criteria, both reviews emphasize the need for stronger longitudinal designs and methodologies to advance neighborhood walkability and obesity research [5, 6].

In describing potential reasons for the attenuation of evidence between objective walkability measures and obesity, Chandrabose et al. [6] cite the uncertain geographic context problem and note less than one-fourth of longitudinal studies use network buffers. Network buffers may better capture walkability-based behaviors [7], and Walk Score® is one example of an objective walkability index based on network distances [8]. Walk Score® is a patented system available in multiple countries that generates a summary walkability score for an address based on population density, street connectivity, and distance to nearby amenities (e.g., grocery stores, coffee shops, restaurants, bars, movie theaters, schools, parks, libraries, book stores, fitness centers, drug stores, hardware stores, clothing stores, music stores) [9]. Research has validated Walk Score® against objective, geographic information systems (GIS)-derived walkability measures at varying spatial scales (400-, 800-, and 1600-m street network buffers, with correlations highest at 1600-m buffers) and metropolitan regions of the United States (US) [10], and has demonstrated its association with perceived proximity to amenities in rural areas [10, 11]. Moreover, Walk Score® offers both a time and cost savings to researchers, health practitioners, policy-makers, and community groups given it is publicly available and it does not require extensive knowledge and technical skills related to GIS data management and processing [9, 12]. Acceptance of Walk Score® has also grown over the years and it has consistently been used across various fields of research in the US [13–15]. This is an important trend as Walk Score® is a policy-relevant BE characteristic and health and social science research calls for greater use of policy-relevant metrics of the BE to foster the translation of active living research into policies and practices addressing chronic disease prevention [16, 17]. Walk Score® is also a consistent measure that can be used across studies. In Canada, researchers have validated Walk Score® along the rural–urban continuum [18]. Canadian studies have capitalized on this validation by examining Walk Score® and obesity across 10 Canadian provinces [19]. However, the largest multi-region study of Walk Score® and obesity among US adults was limited to six urban and suburban communities [20, 21]. Longitudinal, US studies with longer follow-up periods and covering broader geographic regions [6, 21] will improve our understanding of Walk Score® and broaden the generalizability of results.

Across studies with long follow-up periods, it is important to account for residential relocation across time. Comprehensive retention strategies and recording of key dates (residential moves) allow us to calculate exposures with a high degree of accuracy for people who stayed at the same address (stayers) and people who moved (movers) during the study period. The recent systematic review and meta-analysis of the BE and cardio-metabolic health conducted by Chandrabose et al. [6] reports many of the studies on stayers had issues in their research design because participants did not explicitly report residential moves or the studies assumed participants did not move addresses. In studies of health conditions that progress slowly over time (e.g., overweight/obesity and moderate-to-high risk WC), valid and reliable assessment of residential locations is critical for characterizing exposure magnitude and duration within a single place (e.g., neighborhood environment), as well as characterizing the cumulative impact of residential neighborhood environments over time.

The current study will address these limitations using a well-characterized longitudinal cohort. Namely, we use longitudinal data across the US rural–urban continuum to compute a duration-weighted Walk Score® variable and predict the odds of two adiposity-related outcomes (being overweight/obese; having a moderate-to-high risk WC) after controlling for baseline BMI, baseline WC, and other pertinent covariates. We expect the findings will support the walkability-adiposity relationship found in previous literature while addressing important limitations.

## Methods

### Data collection procedures

#### Recruitment

This study used up to 13.3 years of longitudinal data (M (SD) = 9.4 years (1.0 years)) from the REasons for Geographic And Racial Differences in Stroke (REGARDS) study, which is an ongoing observational study of stroke disparities across the contiguous US [22]. REGARDS recruited participants via a commercially available list of mailing addresses and telephone numbers stratified by region-race-sex-age strata. Region was stratified into three categories: Stroke Buckle (coastal plains of North Carolina, South Carolina, and Georgia), Stroke Belt (Alabama, Mississippi, Tennessee, Arkansas, Louisiana, and the rest of North Carolina, South Carolina, and Georgia), and other areas in the continental US. The Stroke Belt concept was coined in 1965 after researchers highlighted the disparate distribution of stroke mortality in the US [23]. Compared to the rest of the US, stroke mortality is higher in the Stroke Belt region and even higher in the Stroke Buckle region [22–24]. Within each of the three region categories (Stroke Buckle, Stroke Belt, and other areas of the continental US), sampling was further stratified by race, sex, and age. Participants needed to speak English, be at least 45 years of age, self-identify their race as White or Black/African American, and not be a current nursing home resident or on a nursing home waiting list. The study excluded those who had cognitive impairment, were undergoing treatment for cancer, or had a medical condition preventing long-term participation. Participants provided written informed consent at baseline and follow-up, and all participating Institutional Review Boards approved the study.

#### Baseline

From February 2003 through October 2007, REGARDS staff collected baseline data on 30,239 enrolled adults. Baseline data collection consisted of a computer-assisted telephone interview (CATI) in which a trained telephone interviewer ascertained demographic, medical history, and health behavior data. Approximately two weeks later, trained personnel conducted a baseline in-home examination consisting of anthropometric measurements, a resting electrocardiogram (ECG), medication inventory, phlebotomy, and urine collection. In-home exams occurred in the morning due to preference for fasted participants. Participants completed and returned additional demographic and risk factor information via self-administered questionnaires.

#### Follow-up

After baseline, REGARDS staff followed up with participants via telephone at six-month intervals to ascertain stroke events. Additionally, from April 2013 to December 2016, trained personnel conducted a second round of in-home examinations. Data collection content and procedures were similar to baseline. Additional details on recruitment, study design, and data collection, handling, and processing are available elsewhere [22].

### Geospatial procedures

Participants provided their residential address at enrollment, confirmed their address at each six-month interval follow-up call, and staff contacted participants via mail once a year to confirm/update participant addresses. Staff also updated addresses through other regular mailings, LexisNexis® technology, and ancillary study contacts. Staff recorded 41,876 addresses from February 2003—September 2017. The significant effort employed in tracking the relocation of participants suggests a high degree of certainty in our ability to assign Walk Score® values to each location participants lived during their time in the study.

Addresses were geocoded using Environmental Systems Research Institute (Esri) ArcGIS® Business Analyst® Desktop 10.5.1 with Esri 2016 Business Analyst® Data used as the reference data. A composite address locator was created using address locators available in Business Analyst® [25]. Only point address and street address matches with a minimum match score of 90 were used in this study [25]. ArcGIS® assigns a match score value based on how well the location found in the reference data matches the participant address being searched [26]. This setting in ArcGIS® allows a researcher to control how closely an address must match its most likely location in the reference data to be considered valid. Esri characterizes a match score of 85 and above as a good match [27]. The study team required a higher level of confidence and selected 90 as the minimum acceptable match score. Staff investigated unmatched records (4%) using manual searches (e.g., Google Maps®, internal notes, and LexisNexis®). After investigation, staff processed new addresses using the address locator and geocoded addresses using the same specifications outlined above. This resulted in 41,004 matched locations (98%) and 872 unmatched locations (2%).

### Measures

#### Neighborhood walkability

An objective measure of neighborhood walkability was calculated using Walk Score® services. Using an address, the Walk Score® methodology analyzes numerous walking routes to nearby amenities (e.g., grocery stores, coffee shops, restaurants, bars, movie theaters, schools, parks, libraries, book stores, fitness centers, drug stores, hardware stores, clothing stores, music stores) and awards points based on distance to amenities [9]. Maximum points are awarded to amenities within a five-minute walk of an address (400 m), while amenities beyond this point are awarded points based on a distance decay function. No points are awarded beyond a 30-min walk (about 2000 m). All geocoded addresses were assigned a Walk Score® value ranging from 0–100 based on neighborhood attributes (population density, street connectivity, and distance to nearby amenities) in 2018 [28]. A duration-weighted Walk Score® value accounted for exposure severity and time at each residence (approximately 28% of participants moved). To create duration-weighted Walk Score® values, we first divided the number of days the participant lived at each address by the participant’s total duration in the study (number of days from baseline to follow-up). This proportion for each address was then multiplied by the corresponding Walk Score® value for that address. For each participant, weighted values were summed to get an overall duration-weighted Walk Score® value (0–100). Walk Score® values were categorically expressed as five categories based on cut-points defined in Walk Score® methodology [8]: Very Car-Dependent (0–24; referent group), Car-Dependent [25–49], Somewhat Walkable (50–69), Very Walkable (70–89), or Walker’s Paradise (90–100). This nomenclature was developed by Walk Score®, provides consistency for describing and characterizing Walk Score® values that fall within a specified range, and is widely used in the literature across countries, fields of study, and populations [8, 28–32].

#### BMI and WC

Trained staff measured participants’ weight, height, and WC following a standardized procedure at both in-home visits. Weight was measured in pounds and ounces (to the nearest quarter of an ounce) using a digital scale. Height was measured in feet and inches (to the nearest quarter of an inch) using an electronic stadiometer [33]. Subjects removed their shoes for both measurements. With the subject standing, WC was measured in inches (to the nearest quarter of an inch) midway between the lowest rib and iliac crest using a tape measure. All measurements were converted to the metric system. BMI was calculated as weight in kilograms divided by height in meters squared. Baseline BMI and WC were treated as continuous covariates. At follow-up, clinically significant cut-points were used for categorization of the adiposity outcomes. Participants were classified into groups based on World Health Organization (WHO) BMI cut-points and further collapsed into two categories: those with a BMI ≥ 25.0 kg/m^2^ were categorized as overweight/obese, while those with a BMI < 25.0 kg/m^2^ were categorized as normal weight/underweight (referent group) [34]. Similarly, at follow-up, participants were classified into groups based on WHO WC cut-points and further collapsed into two categories based on sex. Men with a WC ≥ 94.0 cm and women with a WC ≥ 80.0 cm were considered to have a moderate-to-high risk WC, while participants with WC values falling below their respective, sex-specific WC thresholds were considered to have a low-risk WC (referent group) [35]. Capturing the classification of overweight/obesity and moderate-to-high risk WC is rooted in research highlighting the clinically significant increase in the overall risk of morbidity and mortality for individuals with a BMI ≥ 25.0 kg/m^2^ (includes those who are overweight and obese), and men with a WC ≥ 94.0 cm and women with a WC ≥ 80.0 cm (includes those with moderate and high risk WC) [1, 34–36]. Participants with weight, height, BMI, or WC meeting the following criteria at either time point were excluded: weight ≤ 34.0 kg or ≥ 158.8 kg, height ≤ 91.4 cm or ≥ 228.6 cm, BMI < 13.0 kg/m^2^ or > 55.0 kg/m^2^ [37], or WC < 52.0 cm or ≥ 190.0 cm [38].

#### Individual demographic characteristics

Demographic characteristics were computed using baseline CATI data. Age was calculated using the participant’s birthdate and treated as a continuous variable. Participants’ sex and race were each dichotomous variables; female and Black/African American served as the referent groups. Baseline annual household income was ascertained using an unfolding bracket approach, and responses were collapsed into the following categories: less than $20,000 (referent group); $20,000-$34,999; $35,000-$74,999; $75,000 and above; and refused. At baseline, participants indicated the highest grade or year of school completed using a scale and responses were collapsed into four categories: less than high school (referent group), high school graduate, some college, and college graduate and above. Participants’ baseline marital status was classified as single (never been married), married, divorced/separated, or widowed. Single was the referent group. All demographic characteristics reflected what participants reported at baseline.

#### Individual health characteristics

In line with previous neighborhood walkability-adiposity research, we created and controlled for general health measures at baseline that may impact walking behaviors or weight gain between baseline and follow-up and ultimately adiposity at follow-up. This included baseline comorbidities and health conditions [39–41], baseline smoking status [39–42], and baseline alcohol use [41, 42]. Measures were derived from baseline CATI responses and baseline ECG results. A summary vascular comorbidity variable was created based on baseline presence of hypertension, dyslipidemia, heart disease or myocardial infarction, stroke, diabetes, and/or peripheral artery disease. Two questions at baseline assessed smoking status. Participants were categorized as having never smoked (referent group), being a past smoker, or being a current smoker. Baseline alcohol use status was ascertained using two questions which categorized participants as having never used alcohol (referent group), being a past alcohol user, or being a current alcohol user.

#### Neighborhood socioeconomic status

Geocodes were linked to 2000 US Census Bureau geographies to approximate participants’ neighborhood socioeconomic status (NSES). Block group data on wealth/income, education, and occupation from the 2000 US decennial census were used to characterize NSES. Each block group was assigned a NSES score representing the sum of z-scores of six variables: 1) log of median household income; 2) log of median value of housing units; 3) percentage of households receiving interest, dividend, or net rental income; 4) percentage of adults 25 years of age or older who had completed high school; 5) percentage of adults 25 years of age or older who had completed college; and 6) percentage of employed persons 16 years of age or older in executive, managerial, or professional specialty occupations [43, 44]. Average values for all block groups in the full REGARDS cohort were used as the mean. Analytic sample index values ranged from -14.2—19.0, where higher values represented a higher NSES. Data were collapsed into quartiles, with the lowest quartile serving as the referent group [44]. Prior studies of walkability and adiposity controlled for NSES [39, 42].

### Analyses

Descriptive statistics were generated for all variables. One-way ANOVA tests, Pearson’s chi-square tests, and Fisher’s exact tests examined if covariates differed significantly by Walk Score®. *P*-values < 0.05 suggested at least one group’s proportion or mean value differed significantly from the others. Unadjusted and adjusted multiple logistic regression models examined the association of neighborhood walkability with being overweight/obese at follow-up, as well as the odds of having a moderate-to-high risk WC at follow-up. Adjusted models controlled for individual demographics, individual health characteristics, neighborhood socioeconomic status, and time from baseline to follow-up. Prior empirical work guided covariate selection and all covariates were retained in the model regardless of significance given theory.

Post-estimation Wald tests examined whether categorical variables with more than two categories had coefficients simultaneously equal to zero. Orthogonal polynomial contrasts tested for a linear trend of the neighborhood walkability-adiposity associations. Consistent with other researchers, we tested the robustness of our adjusted BMI-based overweight/obesity model by changing the follow-up BMI measure to a BMI measure calculated using *baseline* height and *follow-up* weight [see Additional File 1] [39]. Theory and previous literature highlighted the need for stratified or moderated analyses [19, 45, 46] and prompted us to construct separate models examining two-way interactions between the duration-weighted neighborhood walkability measure and sex, age, race, and NSES. Findings with a *p*-value < 0.05 were considered significant. Extending seminal work by Cohen [47] in classifying effect sizes using Cohen’s d, odds ratios were assigned qualitative descriptors of effect size following the classification scheme developed by Rosenthal [48]: small effect (OR of about 1.5), medium effect (OR of about 2.5), large effect (OR of about 4.0), very large effect (OR of about 10.0). All analyses were performed in Stata 16.

## Results

Of the 30,239 participants, 56 participants with data anomalies were excluded. Of the remaining 30,183 participants, 16,150 completed the follow-up CATI, in-home exam, or both. At follow-up, about 22% of baseline participants had died, while about 25% declined participation. Differences in death and withdrawal are discussed elsewhere [49]. In general, participants who withdrew or died before follow-up were more likely to be a member of a minority group (e.g., Black/African American, low-income), and more likely to have medical conditions (e.g., diabetes) and poor health behaviors (e.g., current smoker).

**Fig. 1:**
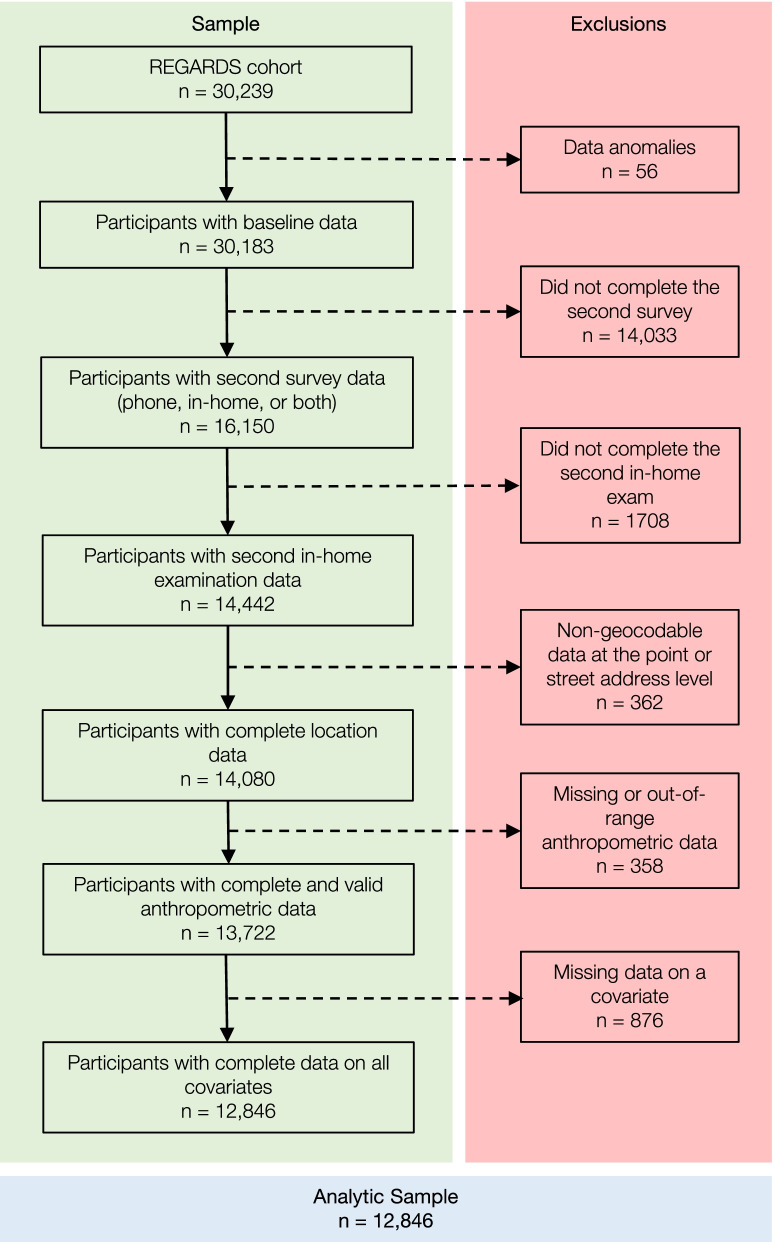
Flowchart depicting the application of exclusion criteria to the full REGARDS cohort

Of participants with follow-up data, additional participants were excluded if they had no in-home examination data (*n* = 1,708), had non-geocodable data at the point or street address level (*n* = 362), had missing or out-of-range anthropometric measurement data (*n* = 358), or had missing data on a covariate (*n* = 876). The analytic sample consisted of 12,846 participants (Fig. 1). An additional file shows descriptives for participants with in-home examination data from both visits who were included versus excluded from the analysis [see Additional File 2]. Briefly, those excluded from the analysis had significantly higher mean BMI values at baseline and higher mean WC values at baseline; were more likely to have a moderate-to-high risk WC at follow-up and less likely to be overweight/obese at follow-up; were less likely to report at baseline that they have ever drank at least one drink per month for one year; and were more likely to live in a more walkable neighborhood, live in a neighborhood with a low SES, be a mover, be younger, have less education, identify as female, Black/African American, unmarried, and low-income. These differences suggest the results may not be generalizable to those who are at the intersection of disadvantage across race, income, education, and sex, as well as those who move often. All other differences were non-significant. Table 1 presents the analytic sample characteristics.

**Table 1:** Sample characteristics by neighborhood walkability

Outcomes	Overall	Very Car-Dependent	Car-Dependent	Somewhat Walkable	Very Walkable	Walker’s Paradise	*P*-value*
	***n* = 12,846**	***n* = 6835**	***n* = 3421**	***n* = 1767**	***n* = 710**	***n* = 113**	
	% or M(SD)	% or M(SD)	% or M(SD)	% or M(SD)	% or M(SD)	% or M(SD)	
*BMI*^*a*^							
Underweight/normal weight	26.09	26.20	25.43	25.18	28.73	37.17	**0.023**
Overweight/obese	73.91	73.80	74.57	74.82	71.27	62.83	
*WC*^*b*^							
Low risk	24.40	24.99	23.24	22.64	28.45	26.55	**0.010**
Moderate-to-high risk	75.60	75.01	76.76	77.36	71.55	73.45	
**Demographic Characteristics**							
*Age (years)*	63.27 (8.39)	62.84 (8.12)	63.72 (8.55)	63.73 (8.83)	63.95 (8.79)	63.49 (9.00)	** < 0.001**
*Sex*							
Female	55.33	52.01	56.91	61.35	62.96	66.37	** < 0.001**
Male	44.67	47.99	43.09	38.65	37.04	33.63	
*Race*							
Black/African American	36.46	25.78	42.59	53.82	63.24	57.52	** < 0.001**
White	63.54	74.22	57.41	46.18	36.76	42.48	
*Income*							
Less than $20,000	12.46	9.71	14.50	16.75	16.76	23.01	** < 0.001**
$20,000 – $34,999	22.25	20.48	23.94	25.30	23.94	19.47	
$35,000 – $74,999	33.73	35.52	32.77	30.05	31.41	26.55	
$75,000 and above	20.89	23.60	17.63	17.88	18.17	20.35	
Refused	10.66	10.68	11.17	10.02	9.72	10.62	
*Education*							
Less than high school	7.62	6.79	8.45	8.83	9.01	5.31	**0.003**
High school graduate	23.54	23.04	24.38	24.05	23.24	22.12	
Some college	26.58	26.88	26.72	26.15	25.49	18.58	
College graduate or above	42.25	43.29	40.46	40.97	42.25	53.98	
*Marital status*							
Single	4.83	2.65	5.29	7.92	12.82	24.78	** < 0.001**
Married	64.75	73.90	58.90	51.56	43.38	29.20	
Divorced/separated	15.79	11.00	19.03	22.92	26.76	27.43	
Widowed	14.62	12.45	16.78	17.60	17.04	18.58	
*Time in study (year)*	9.38 (0.96)	9.33 (0.96)	9.41 (0.93)	9.41 (0.99)	9.49 (0.93)	9.65 (1.03)	** < 0.001**
**Health Characteristics**							
*Baseline BMI (kg/m*^*2*^*)*	29.21 (5.78)	28.89 (5.56)	29.53 (5.95)	29.74 (6.09)	29.45 (6.06)	28.58 (5.47)	** < 0.001**
*Baseline WC (cm)*	95.13 (14.50)	94.71 (14.50)	95.70 (14.47)	95.81 (14.57)	95.13 (14.51)	92.02 (12.74)	** < 0.001**
*Presence of vascular morbidities*							
None	13.33	14.56	11.90	11.60	13.10	11.50	**0.003**
One vascular morbidity	33.58	33.14	33.97	34.18	33.24	41.59	
Two or more vascular morbidities	53.08	52.30	54.14	54.22	53.66	46.90	
*Smoking behaviors*							
Never smoked	48.94	49.92	48.00	47.93	46.90	46.90	**0.001**
Past smoker	40.02	40.07	40.66	38.26	40.70	40.71	
Current smoker	11.04	10.01	11.34	13.81	12.39	12.39	
*Alcohol use*							
Never used alcohol	27.86	28.13	28.88	27.05	23.52	20.35	** < 0.001**
Past alcohol user	15.19	14.05	16.28	17.09	16.62	12.39	
Current alcohol user	56.95	57.82	54.84	55.86	59.86	67.26	
**Contextual Characteristics**							
*NSES*^*c*^							** < 0.001**
Quartile 1 (lowest NSES)	24.50	19.18	29.82	32.48	29.01	31.86	
Quartile 2	25.14	26.28	22.68	25.35	25.07	27.43	
Quartile 3	24.92	25.76	23.53	23.54	28.45	15.04	
Quartile 4 (highest NSES)	25.45	28.78	23.97	18.62	17.46	25.66	

^*^P-values derived from one-way ANOVA tests for continuous variables and Pearson’s chi-square tests or Fisher’s exact tests for categorical variables. *P*-value < 0.05 indicates statistical significance. Significant differences are bolded.

^a^*BMI* body mass index – underweight/normal weight, *BMI* < 25 kg/m^2^; overweight/obese, *BMI* ≥ 25 kg/m^2^

^b^*WC* waist circumference – low: men with a *WC* < 94 cm or women with a *WC* < 80 cm; moderate-to-high: men with a WC ≥ 94 cm or women with a WC ≥ 80 cm

^c^*NSES* neighborhood socioeconomic status

A majority of participants had duration-weighted Walk Score® values in the Very Car-Dependent range (53%) and few participants had values in the Walker’s Paradise range (1%). Nearly three-fourths of participants were overweight/obese and/or had a moderate-to-high risk WC at follow-up. Time elapsed from baseline to follow-up ranged from 5.7 – 13.3 years (M (SD) = 9.4 (1.0)) and approximately 70% of the sample never moved residences from baseline to follow-up (stayers). Across neighborhood walkability types the sample characteristics varied significantly on both outcomes, the exposure, and all covariates.

Table 2 shows unadjusted and adjusted model results for each outcome. In the unadjusted BMI-based model, having a duration-weighted Walk Score® value in the Walker’s Paradise range was associated with significantly lower odds of being overweight/obese at follow-up. Similarly, the adjusted BMI-based model using respective height measurements at each time point indicates Walk Score® is significantly associated with overweight/obesity status at follow-up. The post-estimation test for Walk Score® showed the coefficients were not jointly equal to zero (*χ*^*2*^ (4, *N* = 12,846) = 9.56, *p* = 0.049). There is also a significant downward linear trend in the association between neighborhood walkability and overweight/obesity at follow-up (*χ*^*2*^ (1, *N* = 12,846) = 6.73, *p* = 0.010). The odds ratios decrease with increasing neighborhood walkability suggesting more walkable neighborhoods have larger protective effects on the odds of neighborhood residents being overweight/obese at follow-up. The odds of being overweight/obese at follow-up were 45% lower for residents with Walk Score® values in the Walker’s Paradise range compared to residents with values in the Very Car-Dependent range (OR: 0.55, 95% CI: 0.32—0.93, *p* = 0.026). An odds ratio of this size is considered to be a small effect [48]. Similarly, residents with Walk Score® values in the Very Walkable range had 25% lower odds of being overweight/obese at follow-up relative to residents with values in the Very Car-Dependent range (OR: 0.75, 95% CI: 0.59—0.96, *p* = 0.022). Though significant, an odds ratio of this magnitude is considered to be a small effect at best [48]. No significant interactions emerged by sex, age, race, or NSES. An additional file presents the null interaction results [see Additional File 3]. Adjusted models predicting the odds of being overweight/obese at follow-up were generally similar regardless of whether follow-up height or baseline height was used to calculate follow-up BMI [see Additional File 1].

**Table 2:** Logistic regression models predicting the odds of overweight/obesity^a^ or having a moderate-to-high risk WC^b^ at follow-up

	**Unadjusted BMI Model**^**c**^				**Adjusted BMI Model**^**d**^				**Unadjusted WC Model**^**e**^				**Adjusted WC Model**^**f**^			
	**OR**	**95% CI**	*P*-value*	**χ**^**2**^	**OR**	**95% CI**	*P*-value*	**χ**^**2**^	**OR**	**95% CI**	*P*-value*	**χ**^**2**^	**OR**	**95% CI**	*P*-value*	**χ**^**2**^
**Exposure**																
*Neighborhood walkability*			**0.025**	**11.17**			**0.049**	**9.56**			**0.010**	**13.33**			**0.026**	**11.06**
Very Car-Dependent	1.00	―			1.00	―			1.00	―			1.00	―		
Car-Dependent	1.04	0.95 – 1.14	0.400		0.96	0.84 – 1.09	0.504		1.10	1.00 – 1.21	0.052		0.98	0.86 – 1.10	0.699	
Somewhat Walkable	1.05	0.94 – 1.19	0.384		0.96	0.82 – 1.14	0.676		1.14	1.01 – 1.29	**0.041**		0.99	0.84 – 1.16	0.905	
Very Walkable	0.88	0.74 – 1.05	0.146		0.75	0.59 – 0.96	**0.022**		0.84	0.71 – 1.00	**0.044**		0.69	0.55 – 0.86	**0.001**	
Walker’s Paradise	0.60	0.41 – 0.88	**0.009**		0.55	0.32 – 0.93	**0.026**		0.92	0.60 – 1.40	0.704		0.81	0.48 – 1.37	0.432	
**Demographic Characteristics**																
*Age (year)*					0.96	0.95 – 0.97	** < 0.001**						0.97	0.96 – 0.98	** < 0.001**	
*Sex*																
Female					1.00	―							1.00	―		
Male					1.22	1.08 – 1.38	**0.001**						0.06	0.05 – 0.07	** < 0.001**	
*Race*																
Black/African American					1.00	―							1.00	―		
White					1.10	0.96 – 1.25	0.177						1.17	1.03 – 1.33	**0.013**	
*Income*							0.098	7.84							0.526	3.19
Less than $20,000					1.00	―							1.00	―		
$20,000 – $34,999					1.28	1.04 – 1.57	0.018						1.07	0.87 – 1.31	0.506	
$35,000 – $74,999					1.33	1.08 – 1.64	0.007						1.17	0.95 – 1.44	0.136	
$75,000 and above					1.34	1.06 – 1.71	0.016						1.17	0.93 – 1.47	0.190	
Refused					1.26	0.99 – 1.59	0.058						1.07	0.84 – 1.35	0.596	
*Education*							0.277	3.86							0.523	2.25
Less than high school					1.00	―							1.00	―		
High school graduate					0.84	0.66 – 1.07	0.152						1.04	0.82 – 1.31	0.758	
Some college					0.78	0.62 – 1.00	0.051						0.94	0.74 – 1.19	0.622	
College graduate or above					0.82	0.64 – 1.04	0.107						0.94	0.74 – 1.19	0.588	
*Marital status*							0.997	0.05							0.521	2.25
Single					1.00	―							1.00	―		
Married					0.97	0.74 – 1.29	0.854						1.20	0.92 – 1.57	0.187	
Divorced/separated					0.98	0.73 – 1.33	0.916						1.11	0.83 – 1.48	0.473	
Widowed					0.98	0.73 – 1.34	0.925						1.14	0.85 – 1.54	0.385	
*Time in study (year)*					1.01	0.96 – 1.07	0.616						0.97	0.92 – 1.03	0.298	
**Health Characteristics**																
*Baseline BMI (kg/m*^*2*^*)*					1.72	1.68 – 1.75	** < 0.001**									
*Baseline WC (cm)*													1.17	1.16 – 1.18	** < 0.001**	
*Presence of vascular morbidities*							0.606	1.00							** < 0.001**	**26.30**
None					1.00	―							1.00	―		
One vascular morbidity					1.03	0.88 – 1.20	0.746						1.13	0.97 – 1.31	0.111	
Two or more vascular morbidities					1.08	0.92 – 1.26	0.369						1.42	1.22 – 1.66	** < 0.001**	
*Smoking behaviors*							0.239	2.87							0.783	0.49
Never smoked					1.00	―							1.00			
Past smoker					1.05	0.93 – 1.18	0.446						1.04	0.93 – 1.17	0.499	
Current smoker					0.90	0.75 – 1.07	0.237						1.00	0.84 – 1.20	0.963	
*Alcohol use*							0.205	3.17							0.281	2.54
Never used alcohol					1.00	―							1.00	―		
Past alcohol user					1.18	0.97 – 1.42	0.094						0.90	0.75 – 1.07	0.239	
Current alcohol user					1.02	0.89 – 1.17	0.730						0.90	0.79 – 1.03	0.123	
**Contextual Characteristics**																
*NSES*^*g*^							0.238	4.22							0.313	3.56
Quartile 1 (lowest NSES)					1.00	―							1.00	―		
Quartile 2					1.12	0.95 – 1.32	0.171						0.88	0.75 – 1.03	0.103	
Quartile 3					1.08	0.91 – 1.28	0.378						0.90	0.77 – 1.06	0.211	
Quartile 4 (highest NSES)					0.98	0.82 – 1.17	0.792						0.86	0.73 – 1.02	0.084	

^*^P-value < 0.05 indicates statistical significance. Significant findings are bolded (note significant findings for non-binary categorical variables are based on post-estimation Wald tests).

^a^overweight/obese: body mass index ≥ 25 kg/m^2^

^b^*WC* waist circumference – moderate-to-high risk: men with a *WC* ≥ 94 cm or women with a *WC* ≥ 80 cm

^c^results of test for linear trend (neighborhood walkability and unadjusted body mass index): χ^2^ (1, *N* = 12,846) = 8.66, *p* = 0.003

^d^results of test for linear trend (neighborhood walkability and adjusted body mass index): χ ^2^ (1, *N* = 12,846) = 6.73, *p* = 0.010

^e^results of test for linear trend (neighborhood walkability and unadjusted waist circumference): χ^2^ (1, *N* = 12,846) = 0.98, *p* = 0.322

^f^results of test for linear trend (neighborhood walkability and adjusted waist circumference): χ^2^ (1, *N* = 12,846) = 1.97, *p* = 0.161

^g^*NSES* neighborhood socioeconomic status

In the unadjusted WC model, having a duration-weighted Walk Score® value in the Very Walkable range was significantly associated with having a moderate-to-high risk WC. Surprisingly, in the unadjusted WC model, having a duration-weighted Walk Score® value in the Somewhat Walkable range was positively associated with the outcome—though the direction and significance of the association changed when covariates were added into the model (Table 2). In the adjusted model, the odds of having a moderate-to-high risk WC at follow-up were significantly associated with Walk Score® (Table 2). The post-estimation test for Walk Score® showed the coefficients were not simultaneously equal to zero (*χ*^*2*^ (4, *N* = 12,846) = 11.06, *p* = 0.026). However, unlike the BMI model, there is no significant downward linear trend in the association between neighborhood walkability and the odds of having a moderate-to-high risk WC (*χ*^*2*^ (1, *N* = 12,846) = 1.97, *p* = 0.161). Additionally, only duration-weighted Walk Score® values in the Very Walkable range were significant in the adjusted WC model (OR: 0.69, 95% CI: 0.55—0.86, *p* = 0.001). Participants with duration-weighted Walk Score® values in the Very Walkable range had 31% lower odds of having an unhealthy WC at follow-up compared to participants with values in the Very Car-Dependent range. An odds ratio of this size is considered to be a small effect [48]. No significant interactions emerged by sex, age, race, or NSES. An additional file presents the null interaction results [see Additional File 4].

## Discussion

This study found neighborhood walkability is significantly associated with two, separate characterizations of adiposity (BMI-based overweight/obesity and moderate-to-high risk WC) though the effects are small. In our adjusted overweight/obesity model, the effect sizes corresponding to duration-weighted Walk Score® values in the Very Walkable or Walker’s Paradise range (relative to the Very Car-Dependent range) were similar in magnitude to the effect of baseline income and baseline BMI, respectively. In our adjusted WC model, the effect size corresponding to duration-weighted Walk Score® values in the Very Walkable range (relative to the Very Car-Dependent range) was similar in magnitude to the effect of baseline WC and the presence of vascular morbidities at baseline.

### BMI

A US-based study of military veterans in highly urban counties reported similar results among those living in neighborhoods in the highest walkability quartile compared to the lowest walkability quartile [50]. The authors found living in the highest walkability quartile corresponded to significant decreases in BMI over a six-year period [50]. Hirsch et al. [20] also reported similar BMI results among movers in six US metropolitan areas. Their study found adults who moved to neighborhoods with a 10-point higher Walk Score® saw a resultant decrease in BMI. The aforementioned studies demonstrate objectively-defined neighborhood walkability is associated with significant reductions in BMI over time. Though not directly comparable, the findings are in harmony with our results.

Longitudinal studies of Canadian adults have also shown higher, objective neighborhood walkability is associated with decreased overweight/obesity prevalence [51, 52] and decreases in BMI among men who moved from low-walkable neighborhoods to high-walkable neighborhoods and increases in BMI among men who moved from high-walkable neighborhoods to low-walkable neighborhoods [19]. Wasfi et al. [19] found no significant results among male non-movers nor females. The use of self-reported height and weight in Wasfi et al. [19] may explain the null findings among females. Research shows men are more likely to overreport their height and weight, while women are more likely to overreport their height and underreport their weight [53]. Furthermore, the use of postal code-based Walk Score® values in Wasfi et al. [19] could underestimate the effect, whereas our study used home addresses. Although these results are not directly comparable to our findings, they highlight the predictive power of moving to neighborhoods with Walk Score® values in the upper quartile (Walk Score® values of 70–100; high-walkable neighborhoods) on BMI. This is similar to our study which showed having a duration-weighted Walk Score® value in the Very Walkable range (values ranging from 70–89) and Walker’s Paradise range (values ranging from 90–100), was negatively associated with being overweight/obese at follow-up, but having values in the Car-Dependent range (25–49) or Somewhat Walkable range (50–69) was not significantly associated with overweight/obesity at follow-up.

As one of the largest and longest longitudinal studies of neighborhood walkability and overweight/obesity across the contiguous US, our study advances the literature supporting the longitudinal walkability-adiposity relationship even after controlling for theoretical covariates. The inclusion of a robust BMI measure gathered by trained personnel advances previous research in this area as well. Moreover, our findings show having duration-weighted Walk Score® values in the Very Walkable range or Walker’s Paradise range relative to the Very Car-Dependent range had small but significant associations with being overweight/obese at follow-up, but values falling in the mid-range categories did not.

On the contrary, other longitudinal studies of adults in the US and Canada have found no significant association between neighborhood walkability and BMI-related outcomes [39, 40, 54]. Results reported in these studies tended to be in the expected direction, but did not reach statistical significance. The results may conflict with ours due to at least one of the following: an older sample [39], use of self-reported BMI data [54], and/or use of a spatial scale that potentially represents a less geographically relevant space for estimating neighborhood walkability [39, 40]. Previous research shows after age 60 years, BMI tends to decrease and prevalence of obesity also decreases [55]. Evidence also shows using Euclidean buffers or defining neighborhoods using administrative units may be less theoretically relevant than network buffers [7, 56]. Compared to the sample sizes of the studies which found null effects (*n* = 2,003 [57]; *n* = 1,079 [40]; *n* = 500 [54]), our study also benefited from a very large sample size (*n* = 12,846) allowing us to detect small, but meaningful cumulative effects of neighborhood walkability on a BMI-based outcome.

### WC

The literature examining neighborhood walkability and WC is more limited. In longitudinal studies of neighborhood walkability and WC, the direction of association is mixed. Two studies of neighborhood walkability and WC reported an inverse relationship [42, 58]. However, in two other studies, researchers found neighborhood walkability had a non-significant, positive association with changes in WC over time [21, 52]. Braun et al. [21] hypothesized the non-significant, positive result may be due to the time needed for neighborhood walkability to influence a distal health outcome (e.g., WC), as well as the impact a small sample size (*n* = 538) has on the ability to detect an association. Given Euclidean buffers may be less relevant for estimating neighborhood walkability, the use of Euclidean buffers in Nichani et al. [52] may explain why they found a non-significant, positive association [56]. By using network buffers and up to 13.3 years of longitudinal data from a large cohort, our findings build on this empirical work by demonstrating residents living in neighborhoods with duration-weighted Walk Score® values considered to be Very Walkable have significantly lower odds of having a moderate-to-high risk WC even after accounting for other individual-level characteristics and NSES.

Unlike the overweight/obesity results from the BMI-based model in this study, having a duration-weighted Walk Score® value in the Walker’s Paradise range was not significantly associated with WC at follow-up. The Walker’s Paradise findings are consistent with other researchers who have used a single cohort to examine neighborhood walkability, BMI, and WC and found neighborhood walkability was associated with BMI, but not WC [52]. The mixed findings in the current study regarding the more walkable neighborhood categories may be partly due to the redistribution of body fat that is concurrently happening as people age between baseline and the second assessment in our study (the average age of our analytic sample at baseline is 63.27 years). As people age there are increases in WC even in the absence of weight gain [59]. It is also important to note that postmenopausal women are particularly at risk of central adiposity due to redistributions in weight that occur through the menopausal transition (the average age of menopause is 51) and women make up more than 66% of the Walker’s Paradise group [60, 61]. Moreover, the sample size of the Walker’s Paradise group in the current study was small relative to the other neighborhood walkability categories and thus there was lower power to detect a significant association between our outcomes and those in the Walker’s Paradise group.

### Strengths

An important strength of this study is the large sample size inclusive of stayers and movers across the rural–urban continuum and a range of neighborhood walkability categories. Several prior studies of the BE and cardio-metabolic health are limited in that they did not track participant moves or assumed participants did not move during the follow-up period [6]. Among other factors, two critical methodologies contributing to the large sample and overall strong design of this study include the tracking of relocation dates and the comprehensive and iterative geocoding processes which suggest a high degree of certainty in our ability to assign Walk Score® values to each location participants lived during their time in the study. The fine geographic/spatial scale inherent in a geocode allowed us to approximate neighborhood walkability with precision. This is a strength over other studies which rely on area-level measures rather than fine-grained, individual-level exposures [19, 51].

The objective measure of neighborhood walkability (i.e., Walk Score®) was geocode-based and calculated within network buffers. A recent review highlighted network buffers as a key factor researchers should consider in advancing neighborhood walkability and obesity literature [6]. Duration-weighting this score across time afforded the opportunity to study changes in adiposity longitudinally, while inclusion of theoretical covariates and moderation tests allowed us to isolate the neighborhood walkability-adiposity association. This is critical in our study due to the etiological pathway examined and underlying mechanisms we can expect to be operating across time between neighborhood walkability and adiposity. Both BMI and WC are the result of cumulative exposures over time and the duration-weighted approach in this study captures up to 13.3 years of this cumulative process.

This study used two measures of adiposity measured by trained interviewers. Direct measures of weight, height, and calculated BMI provide a more accurate representation of true anthropometric measurements than self-reported measurements [62]. We also supplement our examination of BMI using a measure of abdominal adiposity (e.g., WC) and encourage future researchers to do the same. Fewer longitudinal studies have examined this association notwithstanding evidence showing weight-based measures (e.g., BMI, WC, waist-to-hip ratio) have both independent and joint effects on health, and despite recommendations or shortcomings noted in previous neighborhood walkability-adiposity research among adults [2, 3, 39, 63–65].

### Limitations

Our study was not without limitations. Walk Score® was calculated based on characteristics of participant neighborhoods in 2018. To the degree the walkability of locations changed over time, there is temporal mismatch between the calculated exposure and the ascription of the exposure to each year. Researchers using 2012 Walk Score® data tested the impact of a similar temporal mismatch and found a minimal and statistically non-significant effect [19]. The null results found by Wasfi et al. [19] suggest the use of 2018 Walk Score® data in our study to represent duration-weighted Walk Score® values prior to that time likely has a small, insignificant effect on our conclusions.

Our exposure measure was also limited to the home neighborhood environment. Our results could misestimate neighborhood walkability associations for individuals who spend more time outside of their residential environment. As suggested by others, future research should expand beyond neighborhood environments to characterize activity spaces [7, 66].

This study was also limited to individuals whom study staff were able to keep in contact with for up to 13.3 years. There is large loss to follow up in this study and the results may not be generalizable to those who are highly transient, change telephone numbers often, lack telephone access, or are at intersecting positions of social disadvantage in terms of race, income, education, and sex. Lastly, this study is subject to potential bias via residential self-selection as lean individuals may self-select into highly walkable environments due to physical activity preferences.

## Conclusions

This observational study advances our understanding of the longitudinal neighborhood walkability-adiposity relationship through integrating various methodological recommendations. This study also has important implications for primary and secondary overweight/obesity prevention. First and foremost, our results suggest low, cumulative neighborhood walkability may be a small but important contributing factor in perpetuating the US obesity epidemic. Underlying this statement is the notion that neighborhood design is a primary prevention tool for obesity. In concert with our previous work with Walk Score® and physical activity in this cohort [28], our findings demonstrate having duration-weighted Walk Score® values in the Very Walkable range or Walker’s Paradise range are significantly associated lower odds of adiposity.

Secondarily, the results point to neighborhood walkability as a potential secondary prevention tool to reduce overweight/obesity. Walk Score® is a publicly available resource available to local government officials, urban planners, community organizations, and other forces combating obesity in the US to identify priority neighborhoods where design changes can be made to systematically target obesity at a neighborhood-level. Overall, neighborhood walkability may be a key factor to consider in US obesity-related policy, advocacy, and programming conversations to help make small but important strides in curtailing the epidemic using a population-driven approach.

## Supplementary Information

**Additional file 1.** Logistic regression models predicting the odds of being overweight/obese using baseline height at follow-up

**Additional file 2.** Descriptive statistics for participants who were included versus excluded from the analytic sample

**Additional file 3.** Logistic regression models predicting the odds of being overweight/obese at follow-up

**Additional file 4.** Logistic regression models predicting the odds of having a moderate-to-high risk wc at follow-up

## Data Availability

The data that support the findings of this study are available from the REasons for Geographic And Racial Differences in Stroke (REGARDS) Coordinating Center at the University of Alabama at Birmingham (UAB) but restrictions apply to the availability of these data, which were used under license for the current study, and so are not publicly available. Data are however available upon reasonable request from the REGARDS Coordinating Center at UAB, regardsadmin@uab.edu.

## Abbreviations

BE: Built environment
BMI: Body mass index
CATI: Computer-assisted telephone interview
ECG: Electrocardiogram
Esri: Environmental Systems Research Institute
NSES: Neighborhood socioeconomic status
REGARDS: REasons for Geographic And Racial Differences in Stroke
US: United States
WC: Waist circumference
WHO: World Health Organization
