# Supplementary material for: A longitudinal examination of objective neighborhood walkability, body mass index, and waist circumference: the REasons for Geographic And Racial Differences in Stroke study
Source: Int J Behav Nutr Phys Act. 2022 Feb 12;19:17. doi: 10.1186/s12966-022-01247-7 (PMC8841052; doi:10.1186/s12966-022-01247-7)
Supplement: Supplementary file 1 — Additional file 1. Logistic regression models predicting the odds of being overweight/obese using baseline height at follow-up [file 12966_2022_1247_MOESM1_ESM.docx]

**ADDITIONAL FILE 1**

| **Table s1. Logistic regression models predicting the odds of being overweight/obese^a^ using baseline height at follow-up** | | | | | | | | |
| --- | --- | --- | --- | --- | --- | --- | --- | --- |
|  | **Unadjusted BMI Model^b^** | | | | **Adjusted BMI Model^c^** | | | |
|  | **OR** | **95% CI** | ***P*-value*** | **χ^2^** | **OR** | **95% CI** | ***P*-value*** | **χ^2^** |
| **Exposure** |  |  |  |  |  |  |  |  |
| *Neighborhood walkability* |  |  | 0.098 | 7.84 |  |  | **0.046** | **9.67** |
| Very Car- Dependent | 1.00 | ― |  |  | 1.00 | ― |  |  |
| Car-Dependent | 1.02 | 0.93 – 1.12 | 0.665 |  | 0.91 | 0.80 – 1.04 | 0.178 |  |
| Somewhat Walkable | 1.02 | 0.91 – 1.14 | 0.751 |  | 0.88 | 0.75 – 1.05 | 0.153 |  |
| Very Walkable | 0.89 | 0.76 – 1.06 | 0.192 |  | 0.74 | 0.58 – 0.95 | **0.018** |  |
| Walker’s Paradise | 0.63 | 0.43 – 0.93 | 0.019 |  | 0.59 | 0.35 – 1.02 | 0.057 |  |
| **Demographic Characteristics** |  |  |  |  |  |  |  |  |
| *Age* |  |  |  |  | 0.94 | 0.94 – 0.95 | **<0.001** |  |
| *Sex* |  |  |  |  |  |  |  |  |
| Female |  |  |  |  | 1.00 | ― |  |  |
| Male |  |  |  |  | 1.29 | 1.15 – 1.46 | **<0.001** |  |
| *Race* |  |  |  |  |  |  |  |  |
| Black/African American |  |  |  |  | 1.00 | ― |  |  |
| White |  |  |  |  | 1.05 | 0.92 – 1.20 | 0.459 |  |
| *Income* |  |  |  |  |  |  | **0.001** | **17.72** |
| Less than $20,000 |  |  |  |  | 1.00 | ― |  |  |
| $20,000 – $34,999 |  |  |  |  | 1.42 | 1.16 – 1.74 | **0.001** |  |
| $35,000 – $74,999 |  |  |  |  | 1.55 | 1.25 – 1.91 | **<0.001** |  |
| $75,000 and above |  |  |  |  | 1.42 | 1.11 – 1.80 | **0.005** |  |
| Refused |  |  |  |  | 1.31 | 1.03 – 1.66 | **0.027** |  |
| *Education* |  |  |  |  |  |  | 0.763 | 1.16 |
| Less than high school |  |  |  |  | 1.00 | ― |  |  |
| High school graduate |  |  |  |  | 0.91 | 0.72 – 1.16 | 0.456 |  |
| Some college |  |  |  |  | 0.88 | 0.69 – 1.12 | 0.289 |  |
| College graduate or above |  |  |  |  | 0.89 | 0.70 – 1.14 | 0.359 |  |
| *Marital status* |  |  |  |  |  |  | 0.938 | 0.41 |
| Single |  |  |  |  | 1.00 | ― |  |  |
| Married |  |  |  |  | 0.94 | 0.71 – 1.24 | 0.653 |  |
| Divorced/separated |  |  |  |  | 0.98 | 0.73 – 1.33 | 0.908 |  |
| Widowed |  |  |  |  | 0.96 | 0.70 – 1.30 | 0.780 |  |
| *Time in study (year)* |  |  |  |  | 0.98 | 0.93 – 1.04 | 0.542 |  |
| **Health Characteristics** |  |  |  |  |  |  |  |  |
| *Baseline BMI (kg/m^2^)* |  |  |  |  | 1.78 | 1.74 – 1.83 | **<0.001** |  |
| *Presence of vascular morbidities* |  |  |  |  |  |  | 0.475 | 1.49 |
| None |  |  |  |  | 1.00 | ― |  |  |
| One vascular morbidity |  |  |  |  | 0.93 | 0.79 – 1.09 | 0.371 |  |
| Two or more vascular morbidities |  |  |  |  | 0.99 | 0.85 – 1.17 | 0.950 |  |
| *Smoking behaviors* |  |  |  |  |  |  | 0.391 | 1.88 |
| Never smoked |  |  |  |  | 1.00 | ― |  |  |
| Past smoker |  |  |  |  | 1.00 | 0.89 – 1.13 | 0.968 |  |
| Current smoker |  |  |  |  | 0.89 | 0.74 – 1.07 | 0.199 |  |
| *Alcohol use* |  |  |  |  |  |  | 0.212 | 3.11 |
| Never used alcohol |  |  |  |  | 1.00 | ― |  |  |
| Past alcohol user |  |  |  |  | 1.19 | 0.98 – 1.43 | 0.080 |  |
| Current alcohol user |  |  |  |  | 1.05 | 0.92 – 1.21 | 0.459 |  |
| **Contextual Characteristics** |  |  |  |  |  |  |  |  |
| *NSES^d^* |  |  |  |  |  |  | 0.221 | 4.40 |
| Quartile 1 (lowest NSES) |  |  |  |  | 1.00 | ― |  |  |
| Quartile 2 |  |  |  |  | 1.00 | 0.85 – 1.18 | 0.961 |  |
| Quartile 3 |  |  |  |  | 1.02 | 0.86 – 1.21 | 0.853 |  |
| Quartile 4 (highest NSES) |  |  |  |  | 0.88 | 0.73 – 1.05 | 0.158 |  |
| ** P*-value < 0.05 indicates statistical significance. Significant findings are bolded (note significant findings for non-binary categorical variables are based on post-estimation Wald tests).  ^a^ *overweight/obese:* body mass index ≥ 25 kg/m^2^  ^b^ results of test for linear trend (neighborhood walkability and unadjusted body mass index): χ^2^ (4, N = 12,846) = 6.80, p = 0.009  ^c^ results of test for linear trend (neighborhood walkability and adjusted body mass index): χ^2^ (4, N = 12,846) = 4.91, p = 0.027  ^d^ *NSES* neighborhood socioeconomic status | | | | | | | | |
|  |  |  |  |  |  |  |  |  |
