# Supplementary material for: A longitudinal examination of objective neighborhood walkability, body mass index, and waist circumference: the REasons for Geographic And Racial Differences in Stroke study
Source: Int J Behav Nutr Phys Act. 2022 Feb 12;19:17. doi: 10.1186/s12966-022-01247-7 (PMC8841052; doi:10.1186/s12966-022-01247-7)
Supplement: Supplementary file 2 — Additional file 2. Descriptive statistics for participants who were included versus excluded from the analytic sample [file 12966_2022_1247_MOESM2_ESM.docx]

**ADDITIONAL FILE 2**

| **Table s2. Descriptive statistics for participants^a^ who were included versus excluded from the analytic sample** | | | | |
| --- | --- | --- | --- | --- |
|  | **Overall**  **n = 14,442**  % or M(SD) | **Included**  **n = 12,846**  % or M(SD) | **Excluded**  **n = 1,596**  % or M(SD) | ***P*-value*** |
|  |  |  |  |  |
| **Exposure** |  |  |  |  |
| *Neighborhood walkability* |  |  |  | **0.006** |
| Very Car-Dependent | 52.56 | 53.21 | 47.37 |  |
| Car-Dependent | 26.64 | 26.63 | 26.75 |  |
| Somewhat Walkable | 13.49 | 13.76 | 11.34 |  |
| Very Walkable | 5.61 | 5.53 | 6.27 |  |
| Walker's Paradise | 0.93 | 0.88 | 1.38 |  |
| Missing | 0.76 | 0.00 | 6.89 |  |
| **Outcomes** |  |  |  |  |
| *BMI^b^* |  |  |  | **0.048** |
| Underweight/normal weight | 25.68 | 26.09 | 22.37 |  |
| Overweight/obese | 73.68 | 73.91 | 71.87 |  |
| Missing | 0.64 | 0.00 | 5.76 |  |
| *WC^c^* |  |  |  | **0.012** |
| Low risk | 24.09 | 24.40 | 21.55 |  |
| Moderate-to-high risk | 75.91 | 75.60 | 78.45 |  |
| **Demographic Characteristics** |  |  |  |  |
| *Age (years)* | 63.21 (8.41) | 63.27 (8.39) | 62.76 (8.55) | **0.023** |
| *Sex* |  |  |  | **<0.001** |
| Female | 56.08 | 55.33 | 62.09 |  |
| Male | 43.92 | 44.67 | 37.91 |  |
| *Race* |  |  |  | **<0.001** |
| Black/African American | 36.93 | 36.46 | 40.73 |  |
| White | 63.07 | 63.54 | 59.27 |  |
| *Income* |  |  |  | **<0.001** |
| Less than $20,000 | 12.97 | 12.46 | 17.04 |  |
| $20,000 – $34,999 | 22.19 | 22.25 | 21.74 |  |
| $35,000 – $74,999 | 33.45 | 33.73 | 31.20 |  |
| $75,000 and above | 20.54 | 20.89 | 17.67 |  |
| Refused | 10.85 | 10.66 | 12.34 |  |
| *Education* |  |  |  | **<0.001** |
| Less than high school | 7.95 | 7.62 | 10.59 |  |
| High school graduate | 23.47 | 23.54 | 22.87 |  |
| Some college | 26.57 | 26.58 | 26.44 |  |
| College graduate or above | 41.99 | 42.25 | 39.85 |  |
| Missing | 0.03 | 0.00 | 0.25 |  |
| *Marital status* |  |  |  | **<0.001** |
| Single | 5.05 | 4.83 | 6.77 |  |
| Married | 63.99 | 64.75 | 57.83 |  |
| Divorced/separated | 16.13 | 15.79 | 18.86 |  |
| Widowed | 14.76 | 14.62 | 15.91 |  |
| Other | 0.07 | 0.00 | 0.63 |  |
| *Residential relocation status* |  |  |  | **<0.001** |
| Stayer | 70.07 | 71.29 | 60.28 |  |
| Mover | 29.93 | 28.71 | 39.72 |  |
| *Time in study (years)* | 9.38 (0.96) | 9.38 (0.96) | 9.36 (1.01) | 0.560 |
| **Health Characteristics** |  |  |  |  |
| *Baseline BMI (kg/m^2^)* | 29.31 (6.02) | 29.21 (5.78) | 30.21 (7.69) | **<0.001** |
| *Baseline WC (cm)* | 95.32 (15.26) | 95.13 (14.50) | 96.99 (20.54) | **<0.001** |
| *Presence of vascular morbidities* |  |  |  | 0.660 |
| None | 12.45 | 13.33 | 5.33 |  |
| One vascular morbidity | 31.44 | 33.58 | 14.22 |  |
| Two or more vascular morbidities | 49.82 | 53.08 | 23.56 |  |
| Missing | 6.29 | 0.00 | 56.89 |  |
| *Smoking behaviors* |  |  |  | 0.660 |
| Never smoked | 48.93 | 48.94 | 48.81 |  |
| Past smoker | 39.79 | 40.02 | 37.91 |  |
| Current smoker | 10.99 | 11.04 | 10.59 |  |
| Missing | 0.30 | 0.00 | 2.69 |  |
| *Alcohol use* |  |  |  | **0.004** |
| Never used alcohol | 28.29 | 27.86 | 31.7 |  |
| Past alcohol user | 15.19 | 15.19 | 15.23 |  |
| Current alcohol user | 56.52 | 56.95 | 53.07 |  |
| **Contextual Characteristics** |  |  |  |  |
| *NSES^d^* |  |  |  | **<0.001** |
| Quartile 1 (lowest NSES) | 24.77 | 24.50 | 26.94 |  |
| Quartile 2 | 24.75 | 25.14 | 21.62 |  |
| Quartile 3 | 24.76 | 24.92 | 23.50 |  |
| Quartile 4 (highest NSES) | 24.75 | 25.45 | 19.17 |  |
| Missing | 0.97 | 0.00 | 8.77 |  |
| ** P*-values derived from one-way ANOVA tests for continuous variables and Pearson’s chi-square tests or Fisher’s exact tests for categorical variables. P-value < 0.05 indicates statistical significance. Significant findings are bolded.  ^a^ Participants needed to have data at both in-home visits (baseline and follow-up)  ^b^ *BMI* body mass index – underweight/normal weight: BMI < 25 kg/m^2^; overweight/obese: BMI ≥ 25 kg/m^2^  ^c^ *WC* waist circumference – low: men with a WC < 94 cm or women with a WC < 80 cm; moderate-to-high: men with a WC ≥ 94 cm or women with a WC ≥ 80 cm  ^d^ *NSES* neighborhood socioeconomic status | | | | |
