# Supplementary material for: A longitudinal examination of objective neighborhood walkability, body mass index, and waist circumference: the REasons for Geographic And Racial Differences in Stroke study
Source: Int J Behav Nutr Phys Act. 2022 Feb 12;19:17. doi: 10.1186/s12966-022-01247-7 (PMC8841052; doi:10.1186/s12966-022-01247-7)

**ADDITIONAL FILE 3**

| **Table s3. Logistic regression models predicting the odds of being overweight/obese^a^ at follow-up** | | | | | | | | | | | | | | | | |
| --- | --- | --- | --- | --- | --- | --- | --- | --- | --- | --- | --- | --- | --- | --- | --- | --- |
|  | **Neighborhood Walkability x Sex Model** | | | | **Neighborhood Walkability x Race Model** | | | | **Neighborhood Walkability x Age Model** | | | | **Neighborhood Walkability x NSES^b^ Model** | | | |
|  | **OR** | **95% CI** | ***P*-value*** | **χ^2^** | **OR** | **95% CI** | ***P*-value*** | **χ^2^** | **OR** | **95% CI** | ***P*-value*** | **χ^2^** | **OR** | **95% CI** | ***P*-value*** | **χ^2^** |
| **Exposure** |  |  |  |  |  |  |  |  |  |  |  |  |  |  |  |  |
| *Neighborhood walkability x sex* |  |  | 0.224 | 5.68 |  |  |  |  |  |  |  |  |  |  |  |  |
| Very Car-Dependent x Female | 1.00 | ― |  |  |  |  |  |  |  |  |  |  |  |  |  |  |
| Very Car-Dependent x Male | 1.00 | ― |  |  |  |  |  |  |  |  |  |  |  |  |  |  |
| Car-Dependent x Female | 1.00 | ― |  |  |  |  |  |  |  |  |  |  |  |  |  |  |
| Car-Dependent x Male | 1.23 | 0.96 – 1.59 | 0.108 |  |  |  |  |  |  |  |  |  |  |  |  |  |
| Somewhat Walkable x Female | 1.00 | ― |  |  |  |  |  |  |  |  |  |  |  |  |  |  |
| Somewhat Walkable x Male | 1.40 | 1.01 – 1.95 | 0.044 |  |  |  |  |  |  |  |  |  |  |  |  |  |
| Very Walkable x Female | 1.00 | ― |  |  |  |  |  |  |  |  |  |  |  |  |  |  |
| Very Walkable x Male | 1.28 | 0.79 – 2.08 | 0.313 |  |  |  |  |  |  |  |  |  |  |  |  |  |
| Walker’s Paradise x Female | 1.00 | ― |  |  |  |  |  |  |  |  |  |  |  |  |  |  |
| Walker’s Paradise x Male | 1.09 | 0.37 – 3.21 | 0.882 |  |  |  |  |  |  |  |  |  |  |  |  |  |
| *Neighborhood walkability x race* |  |  |  |  |  |  | 0.909 | 1.00 |  |  |  |  |  |  |  |  |
| Very Car-Dependent x African American/Black |  |  |  |  | 1.00 | ― |  |  |  |  |  |  |  |  |  |  |
| Very Car-Dependent x White |  |  |  |  | 1.00 | ― |  |  |  |  |  |  |  |  |  |  |
| Car-Dependent x African American/Black |  |  |  |  | 1.00 | ― |  |  |  |  |  |  |  |  |  |  |
| Car-Dependent x White |  |  |  |  | 1.04 | 0.79 – 1.39 | 0.768 |  |  |  |  |  |  |  |  |  |
| Somewhat Walkable x African American/Black |  |  |  |  | 1.00 | ― |  |  |  |  |  |  |  |  |  |  |
| Somewhat Walkable x White |  |  |  |  | 1.17 | 0.83 – 1.66 | 0.365 |  |  |  |  |  |  |  |  |  |
| Very Walkable x African American/Black |  |  |  |  | 1.00 | ― |  |  |  |  |  |  |  |  |  |  |
| Very Walkable x White |  |  |  |  | 0.96 | 0.58 – 1.57 | 0.859 |  |  |  |  |  |  |  |  |  |
| Walker’s Paradise x African American/Black |  |  |  |  | 1.00 | ― |  |  |  |  |  |  |  |  |  |  |
| Walker’s Paradise x White |  |  |  |  | 0.92 | 0.32 – 2.66 | 0.879 |  |  |  |  |  |  |  |  |  |
| *Neighborhood walkability x age* |  |  |  |  |  |  |  |  |  |  | 0.354 | 4.41 |  |  |  |  |
| Very Car-Dependent |  |  |  |  |  |  |  |  | 1.00 | ― |  |  |  |  |  |  |
| Car-Dependent |  |  |  |  |  |  |  |  | 1.00 | 0.98 – 1.01 | 0.903 |  |  |  |  |  |
| Somewhat Walkable |  |  |  |  |  |  |  |  | 1.01 | 0.99 – 1.03 | 0.241 |  |  |  |  |  |
| Very Walkable |  |  |  |  |  |  |  |  | 1.02 | 0.99 – 1.05 | 0.152 |  |  |  |  |  |
| Walker’s Paradise |  |  |  |  |  |  |  |  | 0.97 | 0.91 – 1.03 | 0.369 |  |  |  |  |  |
| *Neighborhood walkability x NSES* |  |  |  |  |  |  |  |  |  |  |  |  |  |  | 0.260 | 14.67 |
| Very Car-Dependent x Quartile 1 (lowest NSES) |  |  |  |  |  |  |  |  |  |  |  |  | 1.00 | ― |  |  |
| Very Car-Dependent x Quartile 2 |  |  |  |  |  |  |  |  |  |  |  |  | 1.00 | ― |  |  |
| Very Car-Dependent x Quartile 3 |  |  |  |  |  |  |  |  |  |  |  |  | 1.00 | ― |  |  |
| Very Car-Dependent x Quartile 4 (highest NSES) |  |  |  |  |  |  |  |  |  |  |  |  | 1.00 | ― |  |  |
| Car-Dependent x Quartile 1 (lowest NSES) |  |  |  |  |  |  |  |  |  |  |  |  | 1.00 | ― |  |  |
| Car-Dependent x Quartile 2 |  |  |  |  |  |  |  |  |  |  |  |  | 1.45 | 0.98 – 2.13 | 0.061 |  |
| Car-Dependent x Quartile 3 |  |  |  |  |  |  |  |  |  |  |  |  | 1.12 | 0.77 – 1.62 | 0.549 |  |
| Car-Dependent x Quartile 4 (highest NSES) |  |  |  |  |  |  |  |  |  |  |  |  | 1.17 | 0.82 – 1.67 | 0.401 |  |
| Somewhat Walkable x Quartile 1 (lowest NSES) |  |  |  |  |  |  |  |  |  |  |  |  | 1.00 | ― |  |  |
| Somewhat Walkable x Quartile 2 |  |  |  |  |  |  |  |  |  |  |  |  | 1.00 | 0.63 – 1.58 | 0.993 |  |
| Somewhat Walkable x Quartile 3 |  |  |  |  |  |  |  |  |  |  |  |  | 1.13 | 0.71 – 1.80 | 0.600 |  |
| Somewhat Walkable x Quartile 4 (highest NSES) |  |  |  |  |  |  |  |  |  |  |  |  | 1.31 | 0.82 – 2.10 | 0.254 |  |
| Very Walkable x Quartile 1 (lowest NSES) |  |  |  |  |  |  |  |  |  |  |  |  | 1.00 | ― |  |  |
| Very Walkable x Quartile 2 |  |  |  |  |  |  |  |  |  |  |  |  | 2.06 | 1.05 – 4.06 | 0.036 |  |
| Very Walkable x Quartile 3 |  |  |  |  |  |  |  |  |  |  |  |  | 1.02 | 0.53 – 1.96 | 0.948 |  |
| Very Walkable x Quartile 4 (highest NSES) |  |  |  |  |  |  |  |  |  |  |  |  | 1.92 | 0.96 – 3.85 | 0.066 |  |
| Walker’s Paradise x Quartile 1 (lowest NSES) |  |  |  |  |  |  |  |  |  |  |  |  | 1.00 | ― |  |  |
| Walker’s Paradise x Quartile 2 |  |  |  |  |  |  |  |  |  |  |  |  | 2.38 | 0.61 – 9.25 | 0.212 |  |
| Walker’s Paradise x Quartile 3 |  |  |  |  |  |  |  |  |  |  |  |  | 2.53 | 0.46 – 13.98 | 0.286 |  |
| Walker’s Paradise x Quartile 4 (highest NSES) |  |  |  |  |  |  |  |  |  |  |  |  | 0.95 | 0.23 – 3.98 | 0.943 |  |
| *Neighborhood walkability* |  |  |  |  |  |  |  |  |  |  |  |  |  |  |  |  |
| Very Car-Dependent | 1.00 | ― |  |  | 1.00 | ― |  |  | 1.00 | ― |  |  | 1.00 | ― |  |  |
| Car-Dependent | 0.86 | 0.72 – 1.03 | 0.107 |  | 0.93 | 0.73 – 1.18 | 0.530 |  | 1.02 | 0.39 – 2.66 | 0.970 |  | 0.81 | 0.62 – 1.07 | 0.143 |  |
| Somewhat Walkable | 0.83 | 0.66 – 1.03 | 0.093 |  | 0.88 | 0.67 – 1.15 | 0.342 |  | 0.47 | 0.14 – 1.58 | 0.223 |  | 0.87 | 0.63 – 1.20 | 0.398 |  |
| Very Walkable | 0.67 | 0.48 – 0.93 | 0.015 |  | 0.76 | 0.54 – 1.07 | 0.114 |  | 0.22 | 0.04 – 1.21 | 0.081 |  | 0.54 | 0.34 – 0.86 | 0.009 |  |
| Walker’s Paradise | 0.52 | 0.27 – 1.01 | 0.055 |  | 0.56 | 0.28 – 1.14 | 0.111 |  | 3.34 | 0.06 – 177.02 | 0.552 |  | 0.37 | 0.15 – 0.95 | 0.039 |  |
| **Demographic Characteristics** |  |  |  |  |  |  |  |  |  |  |  |  |  |  |  |  |
| *Age* | 0.96 | 0.95 – 0.97 | **<0.001** |  | 0.96 | 0.95 – 0.97 | **<0.001** |  | 0.96 | 0.95 – 0.97 | **<0.001** |  | 0.96 | 0.95 – 0.97 | **<0.001** |  |
| *Sex* |  |  |  |  |  |  |  |  |  |  |  |  |  |  |  |  |
| Female | 1.00 | ― |  |  | 1.00 | ― |  |  | 1.00 | ― |  |  | 1.00 | ― |  |  |
| Male | 1.09 | 0.93 – 1.27 | 0.284 |  | 1.22 | 1.08 – 1.38 | **0.001** |  | 1.23 | 1.09 – 1.38 | **0.001** |  | 1.22 | 1.09 – 1.38 | **0.001** |  |
| *Race* |  |  |  |  |  |  |  |  |  |  |  |  |  |  |  |  |
| Black/African American | 1.00 | ― |  |  | 1.00 | ― |  |  | 1.00 | ― |  |  | 1.00 | ― |  |  |
| White | 1.09 | 0.96 – 1.25 | 0.187 |  | 1.06 | 0.88 – 1.28 | 0.552 |  | 1.10 | 0.96 – 1.26 | 0.152 |  | 1.09 | 0.95 – 1.24 | 0.227 |  |
| *Income* |  |  | 0.092 | 7.98 |  |  | 0.100 | 7.78 |  |  | 0.096 | 7.89 |  |  | 0.093 | 7.95 |
| Less than $20,000 | 1.00 | ― |  |  | 1.00 | ― |  |  | 1.00 | ― |  |  | 1.00 | ― |  |  |
| $20,000 – $34,999 | 1.28 | 1.04 – 1.57 | 0.018 |  | 1.28 | 1.04 – 1.57 | 0.018 |  | 1.28 | 1.04 – 1.57 | 0.018 |  | 1.28 | 1.05 – 1.58 | 0.017 |  |
| $35,000 – $74,999 | 1.34 | 1.09 – 1.65 | 0.006 |  | 1.33 | 1.08 – 1.64 | 0.007 |  | 1.34 | 1.08 – 1.65 | 0.007 |  | 1.34 | 1.08 – 1.65 | 0.007 |  |
| $75,000 and above | 1.35 | 1.06 – 1.71 | 0.014 |  | 1.34 | 1.06 – 1.71 | 0.016 |  | 1.34 | 1.06 – 1.71 | 0.015 |  | 1.35 | 1.06 – 1.72 | 0.014 |  |
| Refused | 1.26 | 0.99 – 1.60 | 0.058 |  | 1.26 | 0.99 – 1.59 | 0.059 |  | 1.26 | 0.99 – 1.60 | 0.058 |  | 1.28 | 1.01 – 1.62 | 0.044 |  |
| *Education* |  |  | 0.303 | 3.64 |  |  | 0.278 | 3.85 |  |  | 0.294 | 3.72 |  |  | 0.269 | 3.93 |
| Less than high school | 1.00 | ― |  |  | 1.00 | ― |  |  | 1.00 | ― |  |  | 1.00 | ― |  |  |
| High school graduate | 0.85 | 0.66 – 1.08 | 0.173 |  | 0.84 | 0.66 – 1.07 | 0.148 |  | 0.84 | 0.66 – 1.07 | 0.162 |  | 0.83 | 0.66 – 1.06 | 0.142 |  |
| Some college | 0.79 | 0.62 – 1.01 | 0.058 |  | 0.78 | 0.61 – 1.00 | 0.051 |  | 0.79 | 0.62 – 1.01 | 0.055 |  | 0.78 | 0.61 – 1.00 | 0.048 |  |
| College graduate or above | 0.82 | 0.64 – 1.05 | 0.121 |  | 0.82 | 0.64 – 1.04 | 0.105 |  | 0.82 | 0.64 – 1.05 | 0.115 |  | 0.81 | 0.63 – 1.04 | 0.096 |  |
| *Marital status* |  |  | 0.996 | 0.06 |  |  | 0.998 | 0.04 |  |  | 0.994 | 0.08 |  |  | 0.998 | 0.04 |
| Single | 1.00 | ― |  |  | 1.00 | ― |  |  | 1.00 | ― |  |  | 1.00 | ― |  |  |
| Married | 0.97 | 0.74 – 1.29 | 0.856 |  | 0.98 | 0.74 – 1.29 | 0.865 |  | 0.97 | 0.73 – 1.28 | 0.803 |  | 0.99 | 0.75 – 1.30 | 0.927 |  |
| Divorced/separated | 0.99 | 0.73 – 1.33 | 0.947 |  | 0.98 | 0.73 – 1.33 | 0.917 |  | 0.97 | 0.72 – 1.31 | 0.864 |  | 1.00 | 0.74 – 1.35 | 0.994 |  |
| Widowed | 0.99 | 0.73 – 1.34 | 0.928 |  | 0.99 | 0.73 – 1.34 | 0.935 |  | 0.98 | 0.72 – 1.33 | 0.888 |  | 1.00 | 0.74 – 1.36 | 0.988 |  |
| *Time in study (year)* | 1.02 | 0.96 – 1.08 | 0.566 |  | 1.01 | 0.96 – 1.07 | 0.623 |  | 1.01 | 0.96 – 1.07 | 0.634 |  | 1.02 | 0.96 – 1.08 | 0.517 |  |
| **Health Characteristics** |  |  |  |  |  |  |  |  |  |  |  |  |  |  |  |  |
| *Baseline BMI (kg/m^2^)* | 1.72 | 1.68 – 1.76 | **<0.001** |  | 1.72 | 1.68 – 1.75 | **<0.001** |  | 1.72 | 1.68 – 1.75 | **<0.001** |  | 1.72 | 1.68 – 1.75 | **<0.001** |  |
| *Presence of vascular morbidities* |  |  | 0.590 | 1.06 |  |  | 0.601 | 1.02 |  |  | 0.595 | 1.04 |  |  | 0.650 | 0.86 |
| None | 1.00 | ― |  |  | 1.00 | ― |  |  | 1.00 | ― |  |  | 1.00 | ― |  |  |
| One vascular morbidity | 1.03 | 0.88 – 1.20 | 0.748 |  | 1.03 | 0.88 – 1.20 | 0.739 |  | 1.03 | 0.88 – 1.20 | 0.748 |  | 1.02 | 0.87 – 1.20 | 0.791 |  |
| Two or more vascular morbidities | 1.08 | 0.92 – 1.26 | 0.360 |  | 1.08 | 0.92 – 1.26 | 0.363 |  | 1.08 | 0.92 – 1.26 | 0.363 |  | 1.07 | 0.91 – 1.25 | 0.415 |  |
| *Smoking behaviors* |  |  | 0.238 | 2.87 |  |  | 0.230 | 2.94 |  |  | 0.235 | 2.90 |  |  | 0.223 | 3.00 |
| Never smoked | 1.00 | ― |  |  | 1.00 | ― |  |  | 1.00 | ― |  |  | 1.00 | ― |  |  |
| Past smoker | 1.05 | 0.93 – 1.19 | 0.424 |  | 1.05 | 0.93 – 1.18 | 0.442 |  | 1.05 | 0.93 – 1.18 | 0.456 |  | 1.05 | 0.93 – 1.19 | 0.407 |  |
| Current smoker | 0.90 | 0.75 – 1.08 | 0.248 |  | 0.89 | 0.75 – 1.07 | 0.230 |  | 0.89 | 0.75 – 1.07 | 0.228 |  | 0.90 | 0.75 – 1.08 | 0.242 |  |
| *Alcohol use* |  |  | 0.201 | 3.21 |  |  | 0.205 | 3.17 |  |  | 0.215 | 3.08 |  |  | 0.235 | 2.89 |
| Never used alcohol | 1.00 | ― |  |  | 1.00 | ― |  |  | 1.00 | ― |  |  | 1.00 | ― |  |  |
| Past alcohol user | 1.18 | 0.97 – 1.42 | 0.093 |  | 1.18 | 0.97 – 1.42 | 0.093 |  | 1.17 | 0.97 – 1.42 | 0.098 |  | 1.17 | 0.97 – 1.41 | 0.110 |  |
| Current alcohol user | 1.02 | 0.89 – 1.17 | 0.747 |  | 1.03 | 0.89 – 1.17 | 0.722 |  | 1.02 | 0.89 – 1.17 | 0.722 |  | 1.02 | 0.89 – 1.17 | 0.746 |  |
| **Contextual Characteristics** |  |  |  |  |  |  |  |  |  |  |  |  |  |  |  |  |
| *NSES^b^* |  |  | 0.263 | 3.99 |  |  | 0.244 | 4.17 |  |  | 0.248 | 4.13 |  |  |  |  |
| Quartile 1 (lowest NSES) | 1.00 | ― |  |  | 1.00 | ― |  |  | 1.00 | ― |  |  | 1.00 | ― |  |  |
| Quartile 2 | 1.12 | 0.95 – 1.32 | 0.180 |  | 1.12 | 0.95 – 1.32 | 0.178 |  | 1.12 | 0.95 – 1.32 | 0.182 |  | 1.17 | 0.97 – 1.41 | 0.716 |  |
| Quartile 3 | 1.08 | 0.91 – 1.28 | 0.368 |  | 1.08 | 0.91 – 1.28 | 0.394 |  | 1.07 | 0.91 – 1.27 | 0.406 |  | 1.02 | 0.89 – 1.17 | 0.972 |  |
| Quartile 4 (highest NSES) | 0.98 | 0.82 – 1.17 | 0.820 |  | 0.97 | 0.81 – 1.17 | 0.776 |  | 0.97 | 0.81 – 1.16 | 0.764 |  | 0.87 | 0.69 – 1.10 | 0.235 |  |
| ** P*-value < 0.05 indicates statistical significance. Significant findings are bolded (note significant findings for non-binary categorical variables are based on post-estimation Wald tests).  ^a^ *overweight/obese:* body mass index ≥ 25 kg/m^2^  ^b^ *NSES* neighborhood socioeconomic status | | | | | | | | | | | | | | | | |


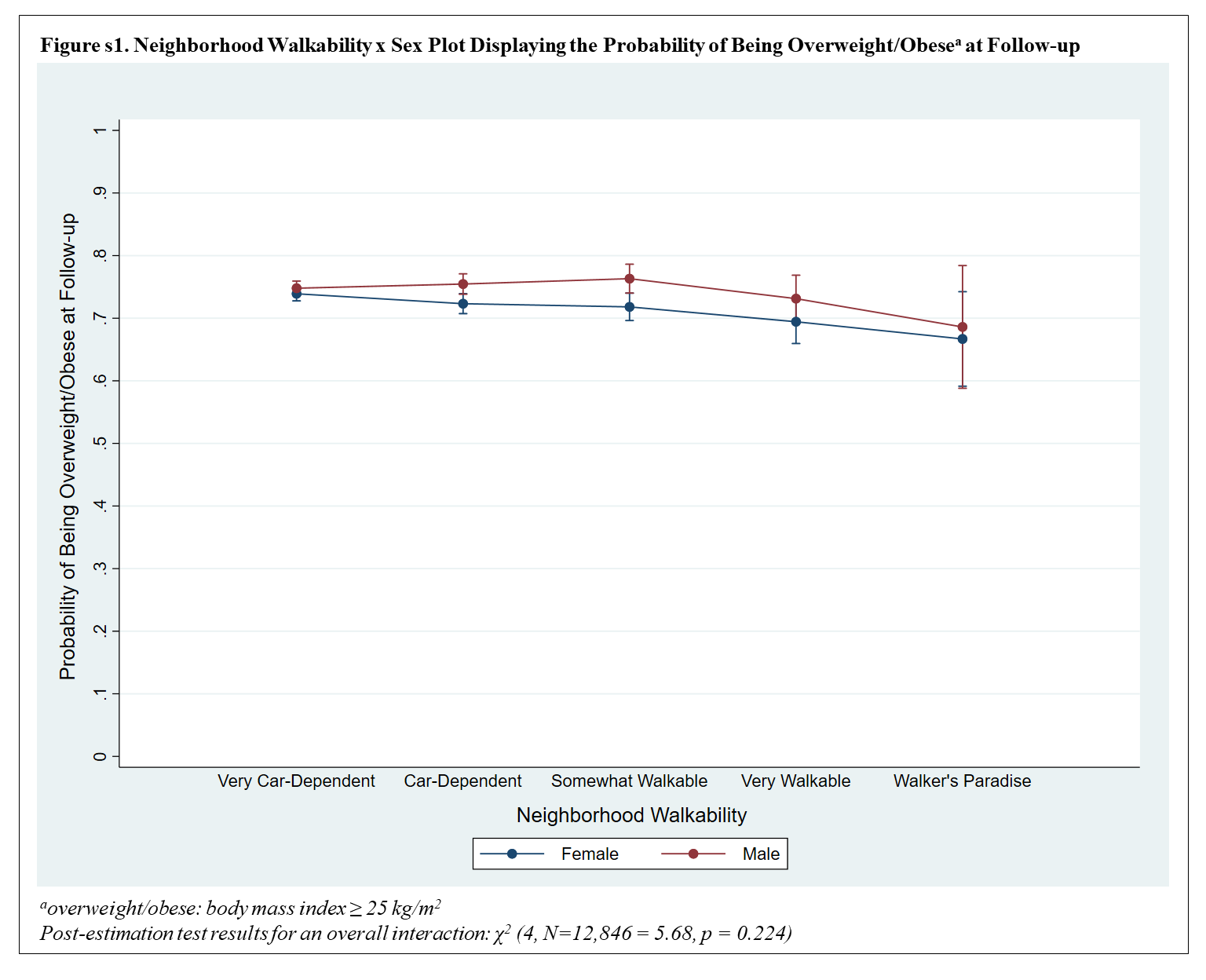

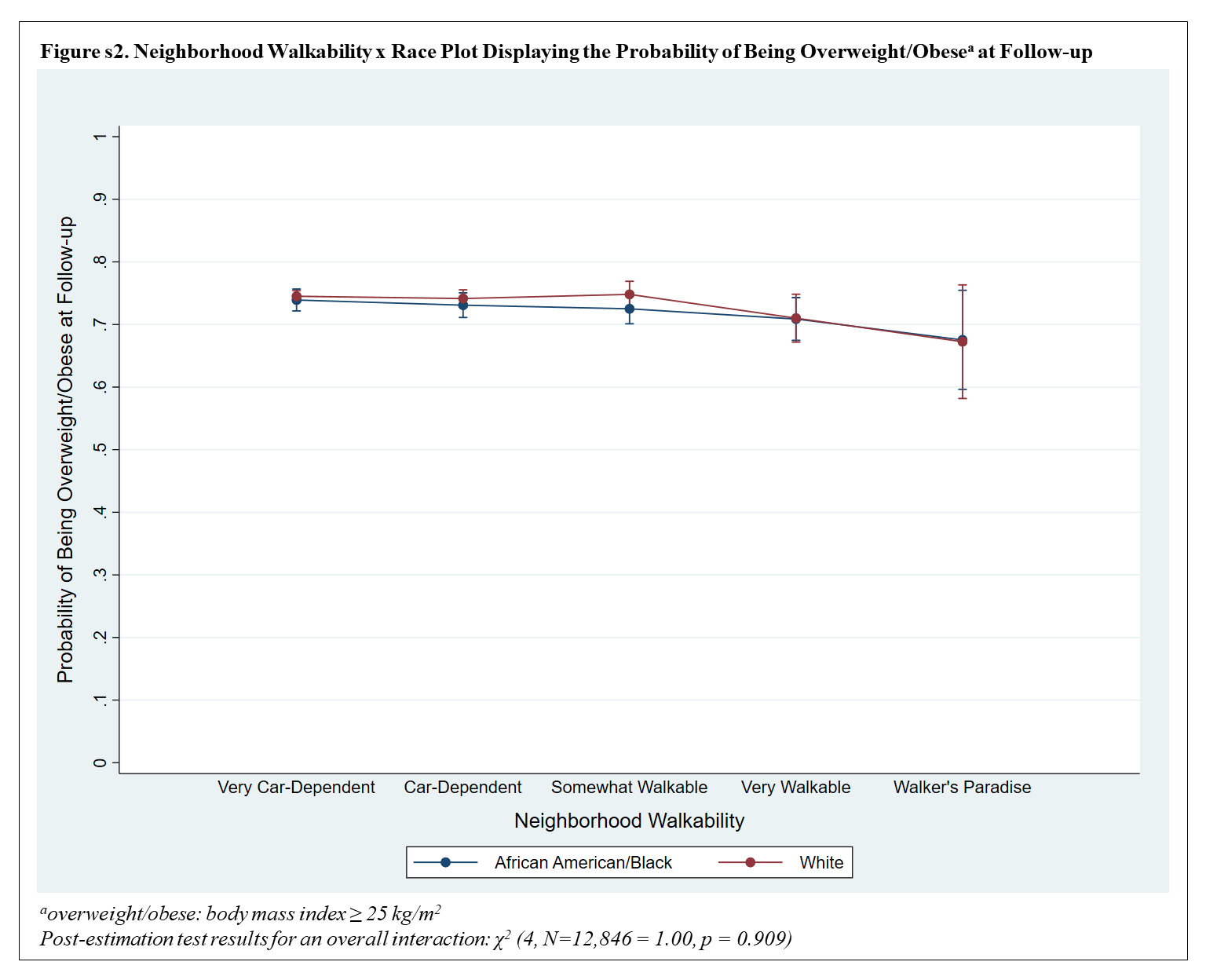

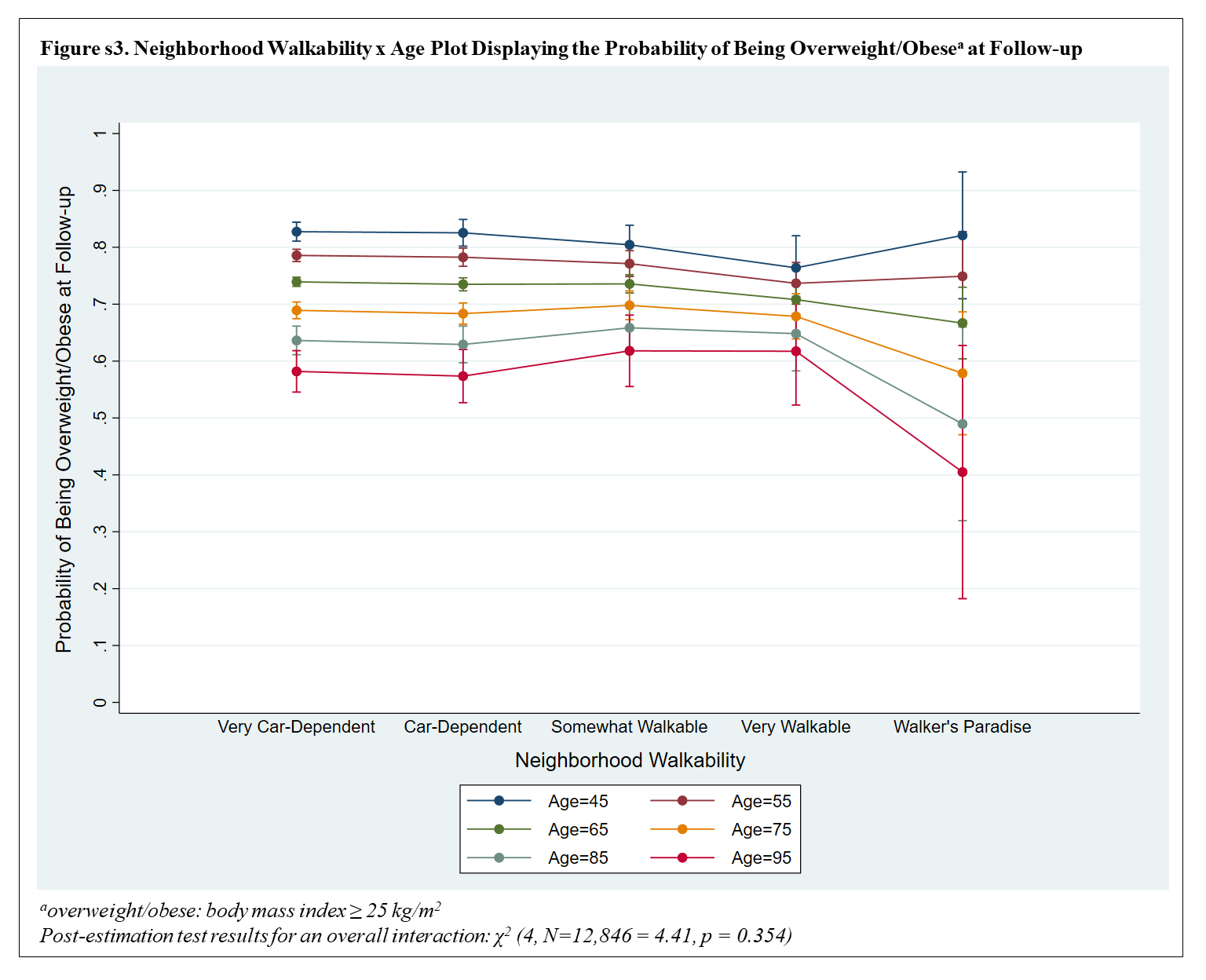

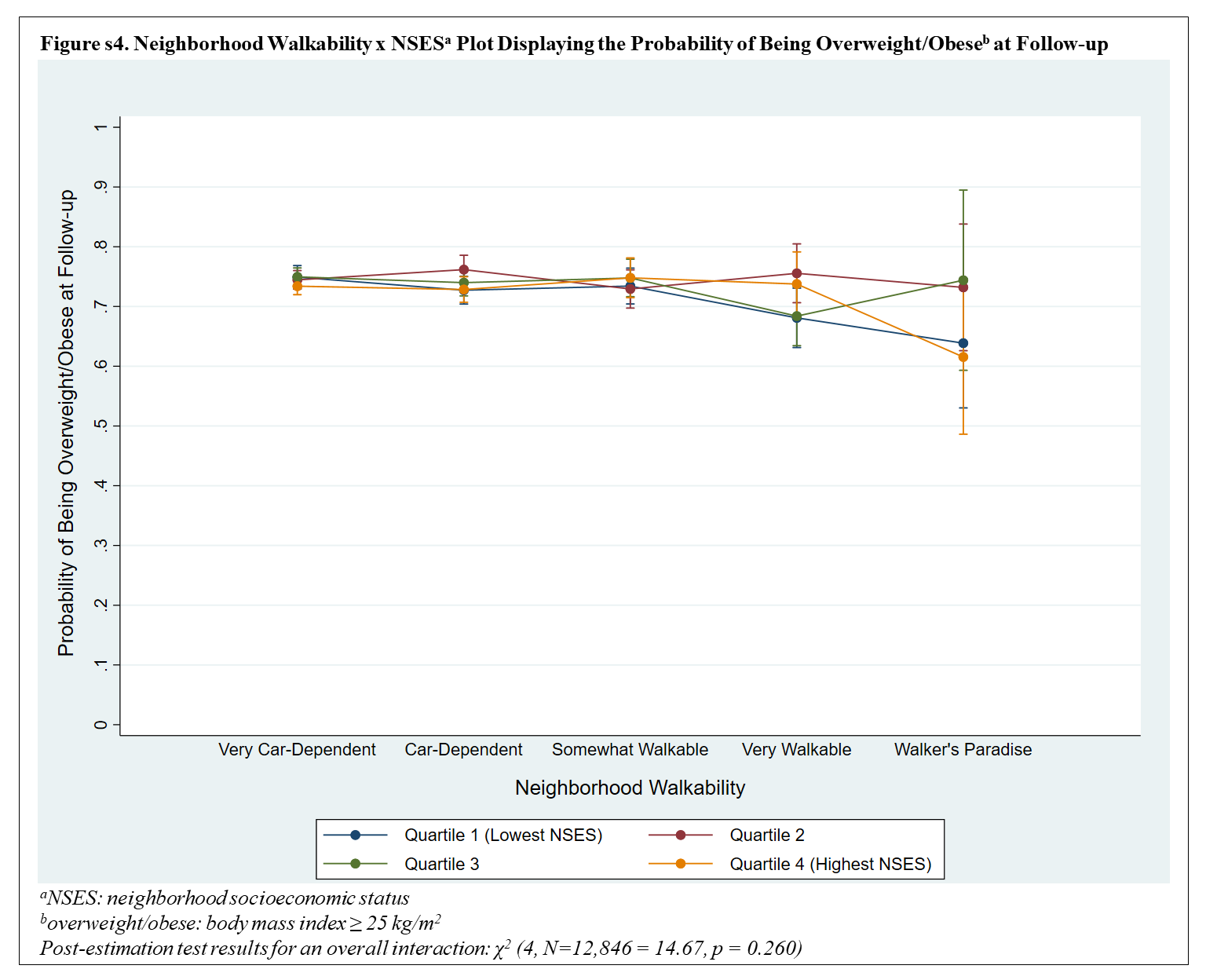

Supplement: Supplementary file 3 — Additional file 3. Logistic regression models predicting the odds of being overweight/obese at follow-up [file 12966_2022_1247_MOESM3_ESM.docx]
