# Supplementary material for: A longitudinal examination of objective neighborhood walkability, body mass index, and waist circumference: the REasons for Geographic And Racial Differences in Stroke study
Source: Int J Behav Nutr Phys Act. 2022 Feb 12;19:17. doi: 10.1186/s12966-022-01247-7 (PMC8841052; doi:10.1186/s12966-022-01247-7)

**ADDITIONAL FILE 4**

| **Table s4. Logistic regression models predicting the odds of having a moderate-to-high risk WC^a^ at follow-up** | | | | | | | | | | | | | | | | |
| --- | --- | --- | --- | --- | --- | --- | --- | --- | --- | --- | --- | --- | --- | --- | --- | --- |
|  | **Neighborhood Walkability x Sex Model** | | | | **Neighborhood Walkability x Race Model** | | | | **Neighborhood Walkability x Age Model** | | | | **Neighborhood Walkability x NSES^b^ Model** | | | |
|  | **OR** | **95% CI** | ***P*-value*** | **χ^2^** | **OR** | **95% CI** | ***P*-value*** | **χ^2^** | **OR** | **95% CI** | ***P*-value*** | **χ^2^** | **OR** | **95% CI** | ***P*-value*** | **χ^2^** |
| **Exposure** |  |  |  |  |  |  |  |  |  |  |  |  |  |  |  |  |
| *Neighborhood walkability x sex* |  |  | 0.535 | 3.14 |  |  |  |  |  |  |  |  |  |  |  |  |
| Very Car-Dependent x Female | 1.00 | ― |  |  |  |  |  |  |  |  |  |  |  |  |  |  |
| Very Car-Dependent x Male | 1.00 | ― |  |  |  |  |  |  |  |  |  |  |  |  |  |  |
| Car-Dependent x Female | 1.00 | ― |  |  |  |  |  |  |  |  |  |  |  |  |  |  |
| Car-Dependent x Male | 0.84 | 0.66 – 1.07 | 0.164 |  |  |  |  |  |  |  |  |  |  |  |  |  |
| Somewhat Walkable x Female | 1.00 | ― |  |  |  |  |  |  |  |  |  |  |  |  |  |  |
| Somewhat Walkable x Male | 1.10 | 0.80 – 1.50 | 0.568 |  |  |  |  |  |  |  |  |  |  |  |  |  |
| Very Walkable x Female | 1.00 | ― |  |  |  |  |  |  |  |  |  |  |  |  |  |  |
| Very Walkable x Male | 1.08 | 0.70 – 1.69 | 0.719 |  |  |  |  |  |  |  |  |  |  |  |  |  |
| Walker’s Paradise x Female | 1.00 | ― |  |  |  |  |  |  |  |  |  |  |  |  |  |  |
| Walker’s Paradise x Male | 1.08 | 0.38 – 3.12 | 0.882 |  |  |  |  |  |  |  |  |  |  |  |  |  |
| *Neighborhood walkability x race* |  |  |  |  |  |  | 0.353 | 4.41 |  |  |  |  |  |  |  |  |
| Very Car-Dependent x African American/Black |  |  |  |  | 1.00 | ― |  |  |  |  |  |  |  |  |  |  |
| Very Car-Dependent x White |  |  |  |  | 1.00 | ― |  |  |  |  |  |  |  |  |  |  |
| Car-Dependent x African American/Black |  |  |  |  | 1.00 | ― |  |  |  |  |  |  |  |  |  |  |
| Car-Dependent x White |  |  |  |  | 1.05 | 0.80 – 1.37 | 0.731 |  |  |  |  |  |  |  |  |  |
| Somewhat Walkable x African American/Black |  |  |  |  | 1.00 | ― |  |  |  |  |  |  |  |  |  |  |
| Somewhat Walkable x White |  |  |  |  | 1.36 | 0.98 – 1.89 | 0.067 |  |  |  |  |  |  |  |  |  |
| Very Walkable x African American/Black |  |  |  |  | 1.00 | ― |  |  |  |  |  |  |  |  |  |  |
| Very Walkable x White |  |  |  |  | 1.33 | 0.84 – 2.12 | 0.226 |  |  |  |  |  |  |  |  |  |
| Walker’s Paradise x African American/Black |  |  |  |  | 1.00 | ― |  |  |  |  |  |  |  |  |  |  |
| Walker’s Paradise x White |  |  |  |  | 1.29 | 0.46 – 3.67 | 0.627 |  |  |  |  |  |  |  |  |  |
| *Neighborhood walkability x age* |  |  |  |  |  |  |  |  |  |  | 0.261 | 5.27 |  |  |  |  |
| Very Car-Dependent |  |  |  |  |  |  |  |  | 1.00 | ― |  |  |  |  |  |  |
| Car-Dependent |  |  |  |  |  |  |  |  | 0.99 | 0.97 – 1.00 | 0.048 |  |  |  |  |  |
| Somewhat Walkable |  |  |  |  |  |  |  |  | 1.00 | 0.99 – 1.02 | 0.723 |  |  |  |  |  |
| Very Walkable |  |  |  |  |  |  |  |  | 1.00 | 0.97 – 1.02 | 0.834 |  |  |  |  |  |
| Walker’s Paradise |  |  |  |  |  |  |  |  | 0.98 | 0.92 – 1.03 | 0.414 |  |  |  |  |  |
| *Neighborhood walkability x NSES* |  |  |  |  |  |  |  |  |  |  |  |  |  |  | 0.124 | 17.72 |
| Very Car-Dependent x Quartile 1 (lowest NSES) |  |  |  |  |  |  |  |  |  |  |  |  | 1.00 | ― |  |  |
| Very Car-Dependent x Quartile 2 |  |  |  |  |  |  |  |  |  |  |  |  | 1.00 | ― |  |  |
| Very Car-Dependent x Quartile 3 |  |  |  |  |  |  |  |  |  |  |  |  | 1.00 | ― |  |  |
| Very Car-Dependent x Quartile 4 (highest NSES) |  |  |  |  |  |  |  |  |  |  |  |  | 1.00 | ― |  |  |
| Car-Dependent x Quartile 1 (lowest NSES) |  |  |  |  |  |  |  |  |  |  |  |  | 1.00 | ― |  |  |
| Car-Dependent x Quartile 2 |  |  |  |  |  |  |  |  |  |  |  |  | 1.07 | 0.74 – 1.53 | 0.734 |  |
| Car-Dependent x Quartile 3 |  |  |  |  |  |  |  |  |  |  |  |  | 1.01 | 0.71 – 1.44 | 0.954 |  |
| Car-Dependent x Quartile 4 (highest NSES) |  |  |  |  |  |  |  |  |  |  |  |  | 1.12 | 0.80 – 1.58 | 0.503 |  |
| Somewhat Walkable x Quartile 1 (lowest NSES) |  |  |  |  |  |  |  |  |  |  |  |  | 1.00 | ― |  |  |
| Somewhat Walkable x Quartile 2 |  |  |  |  |  |  |  |  |  |  |  |  | 0.84 | 0.54 – 1.30 | 0.432 |  |
| Somewhat Walkable x Quartile 3 |  |  |  |  |  |  |  |  |  |  |  |  | 1.42 | 0.91 – 2.24 | 0.124 |  |
| Somewhat Walkable x Quartile 4 (highest NSES) |  |  |  |  |  |  |  |  |  |  |  |  | 1.65 | 1.05 – 2.58 | 0.031 |  |
| Very Walkable x Quartile 1 (lowest NSES) |  |  |  |  |  |  |  |  |  |  |  |  | 1.00 | ― |  |  |
| Very Walkable x Quartile 2 |  |  |  |  |  |  |  |  |  |  |  |  | 0.68 | 0.36 – 1.30 | 0.244 |  |
| Very Walkable x Quartile 3 |  |  |  |  |  |  |  |  |  |  |  |  | 0.72 | 0.39 – 1.32 | 0.291 |  |
| Very Walkable x Quartile 4 (highest NSES) |  |  |  |  |  |  |  |  |  |  |  |  | 0.81 | 0.41 – 1.59 | 0.541 |  |
| Walker’s Paradise x Quartile 1 (lowest NSES) |  |  |  |  |  |  |  |  |  |  |  |  | 1.00 | ― |  |  |
| Walker’s Paradise x Quartile 2 |  |  |  |  |  |  |  |  |  |  |  |  | 2.08 | 0.46 – 9.48 | 0.345 |  |
| Walker’s Paradise x Quartile 3 |  |  |  |  |  |  |  |  |  |  |  |  | 0.44 | 0.09 – 2.02 | 0.288 |  |
| Walker’s Paradise x Quartile 4 (highest NSES) |  |  |  |  |  |  |  |  |  |  |  |  | 1.18 | 0.28 – 4.91 | 0.822 |  |
| *Neighborhood walkability* |  |  |  |  |  |  |  |  |  |  |  |  |  |  |  |  |
| Very Car-Dependent | 1.00 | ― |  |  | 1.00 | ― |  |  | 1.00 | ― |  |  | 1.00 | ― |  |  |
| Car-Dependent | 1.07 | 0.89 – 1.29 | 0.447 |  | 0.93 | 0.75 – 1.17 | 0.551 |  | 2.40 | 0.98 – 5.89 | 0.056 |  | 0.92 | 0.71 – 1.20 | 0.552 |  |
| Somewhat Walkable | 0.95 | 0.75 – 1.19 | 0.636 |  | 0.82 | 0.64 – 1.06 | 0.131 |  | 0.80 | 0.26 – 2.53 | 0.709 |  | 0.84 | 0.61 – 1.16 | 0.289 |  |
| Very Walkable | 0.66 | 0.48 – 0.91 | 0.010 |  | 0.59 | 0.43 – 0.81 | 0.001 |  | 0.81 | 0.16 – 4.07 | 0.801 |  | 0.85 | 0.54 – 1.36 | 0.505 |  |
| Walker’s Paradise | 0.79 | 0.40 – 1.56 | 0.492 |  | 0.70 | 0.34 – 1.45 | 0.333 |  | 3.64 | 0.09 – 140.83 | 0.489 |  | 0.75 | 0.28 – 2.01 | 0.565 |  |
| **Demographic Characteristics** |  |  |  |  |  |  |  |  |  |  |  |  |  |  |  |  |
| *Age* | 0.97 | 0.96 – 0.98 | **<0.001** |  | 0.97 | 0.96 – 0.98 | **<0.001** |  | 0.97 | 0.97 – 0.98 | **<0.001** |  | 0.97 | 0.96 – 0.98 | **<0.001** |  |
| *Sex* |  |  |  |  |  |  |  |  |  |  |  |  |  |  |  |  |
| Female | 1.00 | ― |  |  | 1.00 | ― |  |  | 1.00 | ― |  |  | 1.00 | ― |  |  |
| Male | 0.06 | 0.05 – 0.08 | **<0.001** |  | 0.06 | 0.05 – 0.07 | **<0.001** |  | 0.06 | 0.05 – 0.07 | **<0.001** |  | 0.06 | 0.05 – 0.07 | **<0.001** |  |
| *Race* |  |  |  |  |  |  |  |  |  |  |  |  |  |  |  |  |
| Black/African American | 1.00 |  |  |  | 1.00 | ― |  |  | 1.00 | ― |  |  | 1.00 | ― |  |  |
| White | 1.17 | 1.03 – 1.33 | **0.014** |  | 1.08 | 0.90 – 1.28 | 0.406 |  | 1.18 | 1.04 – 1.33 | **0.011** |  | 1.15 | 1.02 – 1.31 | **0.027** |  |
| *Income* |  |  | 0.517 | 3.25 |  |  | 0.530 | 3.17 |  |  | 0.522 | 3.22 |  |  | 0.547 | 3.07 |
| Less than $20,000 | 1.00 | ― |  |  | 1.00 | ― |  |  | 1.00 | ― |  |  | 1.00 | ― |  |  |
| $20,000 – $34,999 | 1.07 | 0.87 – 1.31 | 0.502 |  | 1.07 | 0.87 – 1.31 | 0.515 |  | 1.07 | 0.87 – 1.31 | 0.500 |  | 1.06 | 0.87 – 1.31 | 0.548 |  |
| $35,000 – $74,999 | 1.17 | 0.95 – 1.44 | 0.131 |  | 1.17 | 0.95 – 1.44 | 0.140 |  | 1.17 | 0.95 – 1.44 | 0.134 |  | 1.17 | 0.95 – 1.43 | 0.146 |  |
| $75,000 and above | 1.17 | 0.93 – 1.48 | 0.185 |  | 1.17 | 0.93 – 1.47 | 0.189 |  | 1.17 | 0.93 – 1.48 | 0.182 |  | 1.16 | 0.92 – 1.47 | 0.211 |  |
| Refused | 1.07 | 0.84 – 1.35 | 0.582 |  | 1.07 | 0.84 – 1.35 | 0.599 |  | 1.07 | 0.84 – 1.35 | 0.579 |  | 1.07 | 0.84 – 1.35 | 0.592 |  |
| *Education* |  |  | 0.515 | 2.29 |  |  | 0.497 | 2.38 |  |  | 0.541 | 2.15 |  |  | 0.533 | 2.19 |
| Less than high school | 1.00 | ― |  |  | 1.00 | ― |  |  | 1.00 | ― |  |  | 1.00 | ― |  |  |
| High school graduate | 1.04 | 0.82 – 1.31 | 0.761 |  | 1.03 | 0.82 – 1.31 | 0.781 |  | 1.03 | 0.82 – 1.31 | 0.779 |  | 1.04 | 0.82 – 1.31 | 0.748 |  |
| Some college | 0.94 | 0.74 – 1.19 | 0.607 |  | 0.94 | 0.74 – 1.19 | 0.619 |  | 0.94 | 0.74 – 1.19 | 0.618 |  | 0.95 | 0.75 – 1.20 | 0.654 |  |
| College graduate or above | 0.94 | 0.74 – 1.19 | 0.587 |  | 0.93 | 0.73 – 1.18 | 0.537 |  | 0.94 | 0.74 – 1.19 | 0.584 |  | 0.94 | 0.74 – 1.19 | 0.595 |  |
| *Marital status* |  |  | 0.513 | 2.30 |  |  | 0.495 | 2.39 |  |  | 0.472 | 2.52 |  |  | 0.538 | 2.17 |
| Single | 1.00 | ― |  |  | 1.00 | ― |  |  | 1.00 | ― |  |  | 1.00 | ― |  |  |
| Married | 1.20 | 0.92 – 1.57 | 0.189 |  | 1.21 | 0.92 – 1.58 | 0.166 |  | 1.21 | 0.93 – 1.58 | 0.162 |  | 1.19 | 0.91 – 1.56 | 0.203 |  |
| Divorced/separated | 1.11 | 0.83 – 1.48 | 0.481 |  | 1.12 | 0.84 – 1.50 | 0.426 |  | 1.12 | 0.84 – 1.49 | 0.450 |  | 1.10 | 0.83 – 1.47 | 0.503 |  |
| Widowed | 1.13 | 0.84 – 1.53 | 0.409 |  | 1.15 | 0.85 – 1.55 | 0.363 |  | 1.16 | 0.86 – 1.56 | 0.339 |  | 1.14 | 0.84 – 1.53 | 0.408 |  |
| *Time in study (year)* | 0.97 | 0.92 – 1.03 | 0.302 |  | 0.97 | 0.92 – 1.03 | 0.310 |  | 0.97 | 0.92 – 1.03 | 0.313 |  | 0.97 | 0.92 – 1.03 | 0.323 |  |
| **Health Characteristics** |  |  |  |  |  |  |  |  |  |  |  |  |  |  |  |  |
| *Baseline WC (cm)* | 1.17 | 1.16 – 1.18 | **<0.001** |  | 1.17 | 1.16 – 1.18 | **<0.001** |  | 1.17 | 1.16 – 1.18 | **<0.001** |  | 1.17 | 1.16 – 1.18 | **<0.001** |  |
| *Presence of vascular morbidities* |  |  | **<0.001** | **26.76** |  |  | **<0.001** | **26.58** |  |  | **<0.001** | **26.43** |  |  | **<0.001** | **25.71** |
| None | 1.00 | ― |  |  | 1.00 | ― |  |  | 1.00 | ― |  |  | 1.00 | ― |  |  |
| One vascular morbidity | 1.13 | 0.97 – 1.31 | 0.106 |  | 1.13 | 0.97 – 1.31 | 0.106 |  | 1.13 | 0.97 – 1.31 | 0.110 |  | 1.13 | 0.97 – 1.31 | 0.119 |  |
| Two or more vascular morbidities | 1.43 | 1.23 – 1.67 | **<0.001** |  | 1.43 | 1.23 – 1.66 | **<0.001** |  | 1.43 | 1.22 – 1.66 | **<0.001** |  | 1.42 | 1.22 – 1.65 | **<0.001** |  |
| *Smoking behaviors* |  |  | 0.775 | 0.51 |  |  | 0.766 | 0.53 |  |  | 0.764 | 0.54 |  |  | 0.796 | 0.46 |
| Never smoked | 1.00 | ― |  |  | 1.00 | ― |  |  | 1.00 | ― |  |  | 1.00 | ― |  |  |
| Past smoker | 1.04 | 0.93 – 1.17 | 0.491 |  | 1.04 | 0.93 – 1.17 | 0.485 |  | 1.04 | 0.93 – 1.17 | 0.481 |  | 1.04 | 0.93 – 1.16 | 0.519 |  |
| Current smoker | 1.00 | 0.84 – 1.20 | 0.970 |  | 1.00 | 0.84 – 1.19 | 0.980 |  | 1.00 | 0.84 – 1.20 | 0.977 |  | 1.00 | 0.84 – 1.19 | 0.988 |  |
| *Alcohol use* |  |  | 0.277 | 2.57 |  |  | 0.266 | 2.65 |  |  | 0.294 | 2.45 |  |  | 0.295 | 2.45 |
| Never used alcohol | 1.00 | ― |  |  | 1.00 | ― |  |  | 1.00 | ― |  |  | 1.00 | ― |  |  |
| Past alcohol user | 0.90 | 0.75 – 1.07 | 0.238 |  | 0.90 | 0.75 – 1.07 | 0.235 |  | 0.90 | 0.75 – 1.07 | 0.243 |  | 0.90 | 0.76 – 1.08 | 0.266 |  |
| Current alcohol user | 0.90 | 0.79 – 1.03 | 0.121 |  | 0.90 | 0.79 – 1.03 | 0.115 |  | 0.90 | 0.79 – 1.03 | 0.132 |  | 0.90 | 0.79 – 1.03 | 0.127 |  |
| **Contextual Characteristics** |  |  |  |  |  |  |  |  |  |  |  |  |  |  |  |  |
| *NSES^a^* |  |  | 0.309 | 3.59 |  |  | 0.305 | 3.62 |  |  | 0.297 | 3.69 |  |  |  |  |
| Quartile 1 (lowest NSES) | 1.00 | ― |  |  | 1.00 | ― |  |  | 1.00 | ― |  |  | 1.00 | ― |  |  |
| Quartile 2 | 0.88 | 0.75 – 1.03 | 0.100 |  | 0.88 | 0.75 – 1.02 | 0.098 |  | 0.88 | 0.75 – 1.03 | 0.101 |  | 0.90 | 0.72 – 1.12 | 0.328 |  |
| Quartile 3 | 0.90 | 0.77 – 1.06 | 0.209 |  | 0.90 | 0.77 – 1.06 | 0.209 |  | 0.90 | 0.77 – 1.06 | 0.204 |  | 0.88 | 0.71 – 1.10 | 0.269 |  |
| Quartile 4 (highest NSES) | 0.86 | 0.73 – 1.02 | 0.084 |  | 0.86 | 0.73 – 1.02 | 0.083 |  | 0.86 | 0.72 – 1.02 | 0.076 |  | 0.80 | 0.65 – 1.00 | 0.052 |  |
| ** P*-value < 0.05 indicates statistical significance. Significant findings are bolded (note significant findings for non-binary categorical variables are based on post-estimation Wald tests).  ^a^ *WC* waist circumference – moderate-to-high risk: men with a WC ≥ 94 cm or women with a WC ≥ 80 cm  ^b^ *NSES* neighborhood socioeconomic status | | | | | | | | | | | | | | | | |


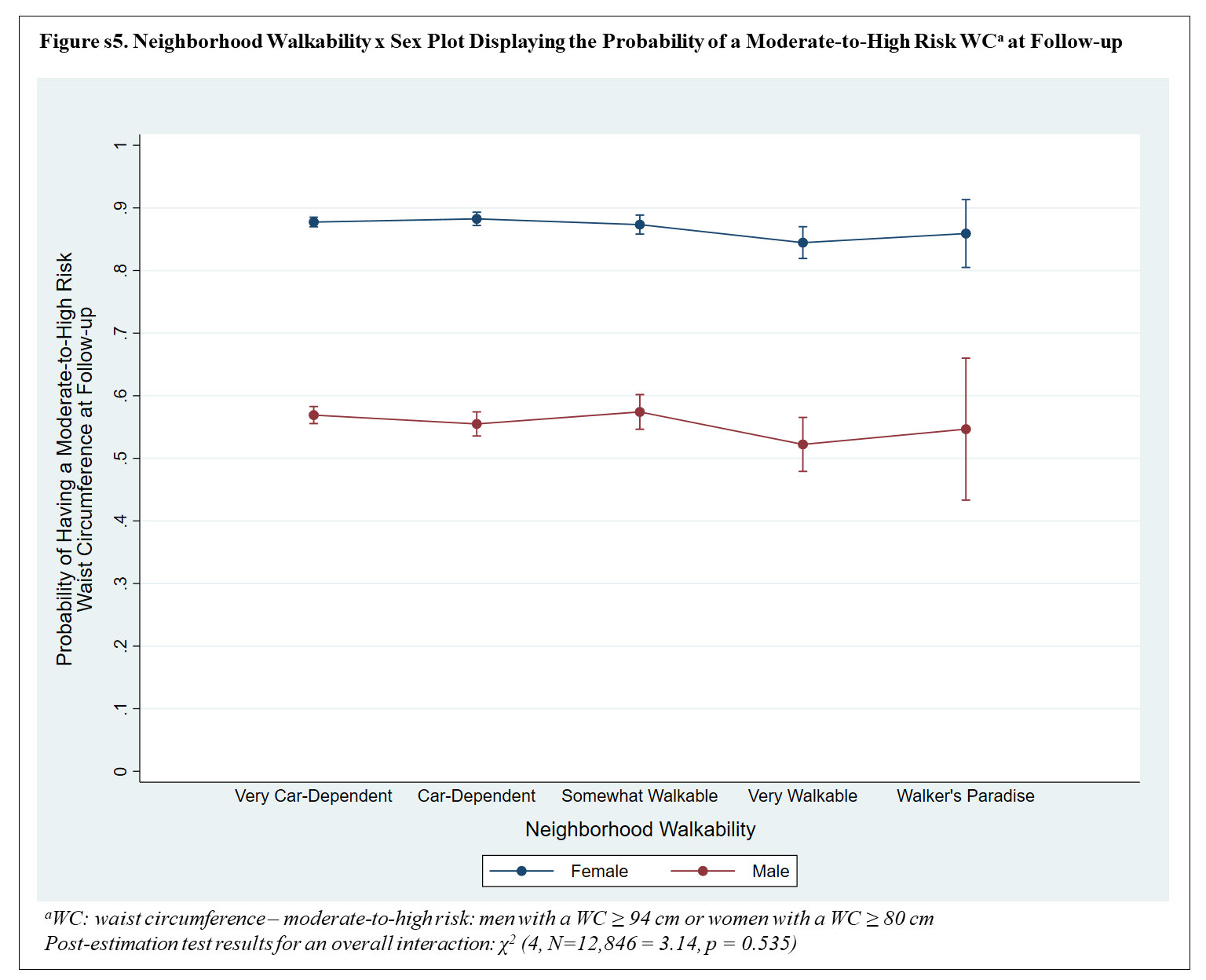

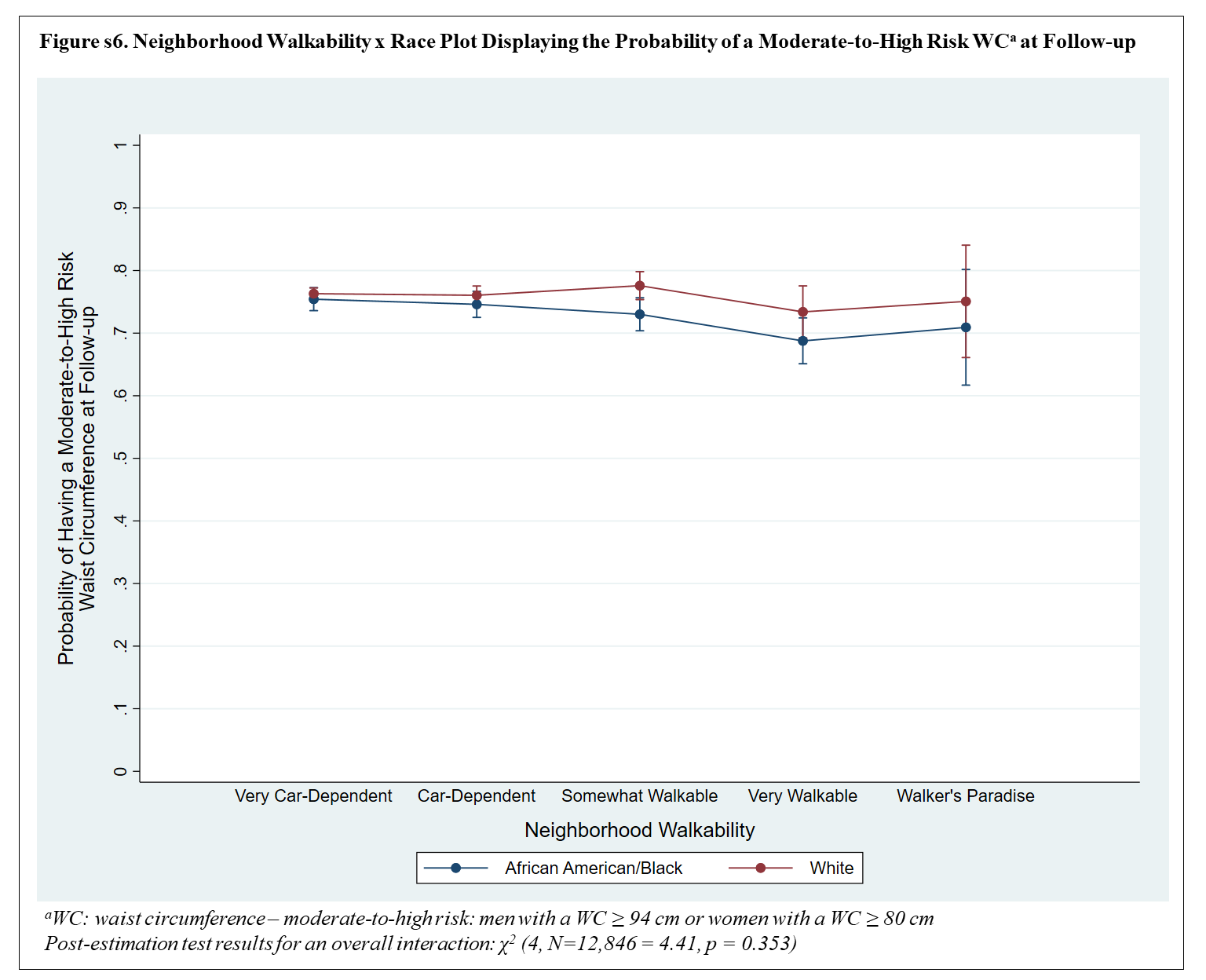

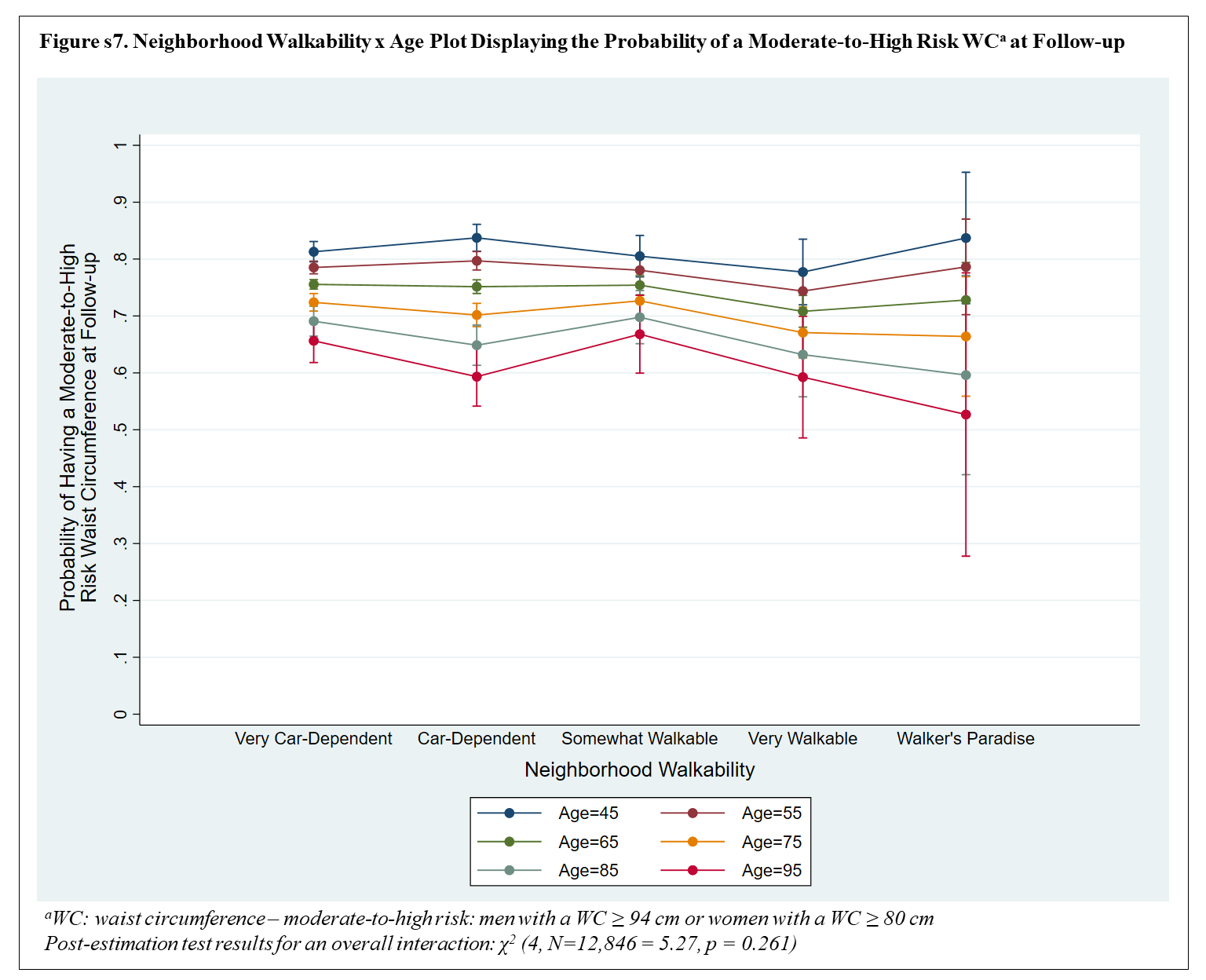

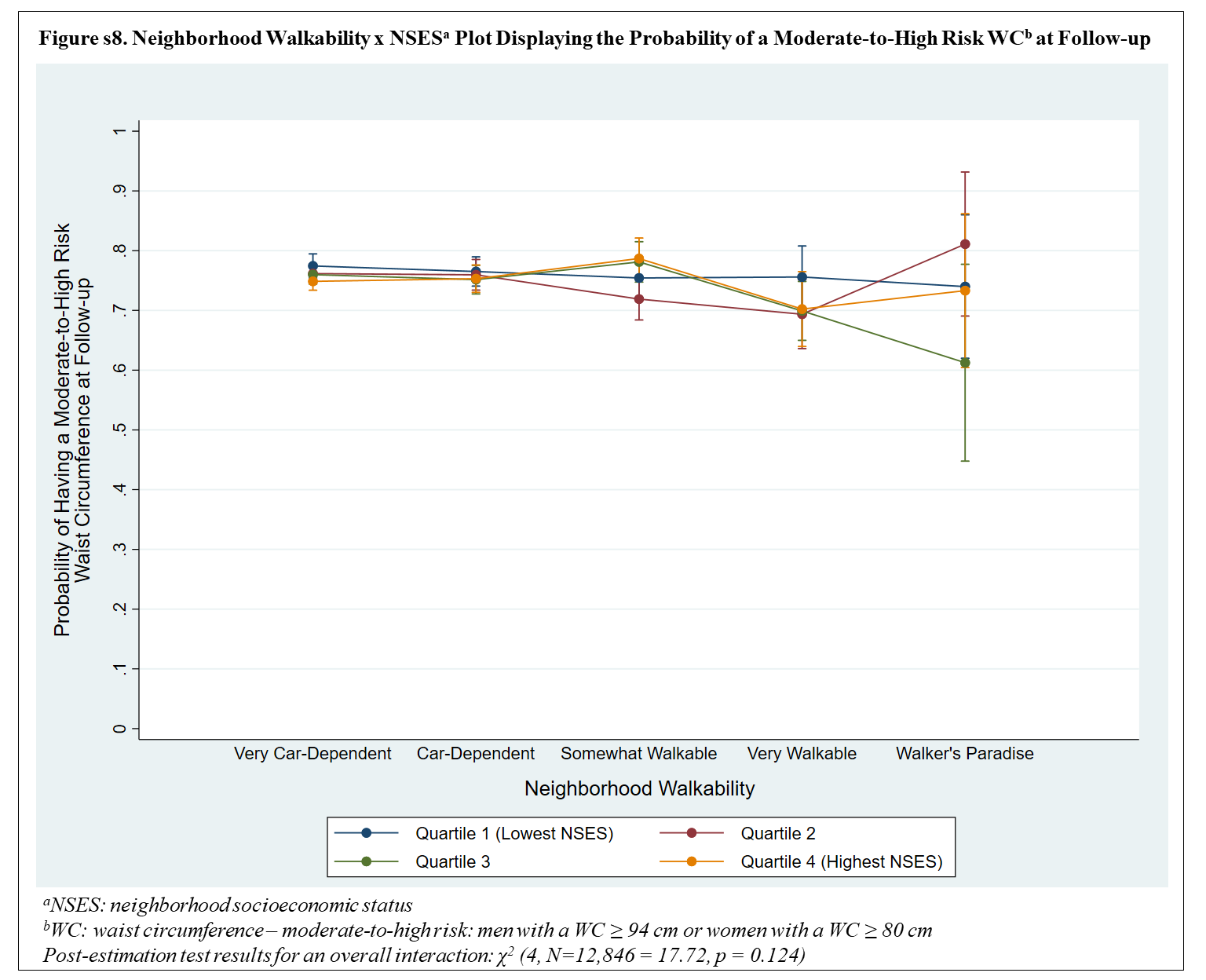

Supplement: Supplementary file 4 — Additional file 4. Logistic regression models predicting the odds of having a moderate-to-high risk wc at follow-up [file 12966_2022_1247_MOESM4_ESM.docx]
